# A Geometric Interpretation of Kinetic Zone Diagrams in Electrochemistry

**DOI:** 10.1021/jacs.4c13271

**Published:** 2024-12-04

**Authors:** Nicolas Plumeré, Ben A. Johnson

**Affiliations:** Technical University of Munich (TUM), Campus Straubing for Biotechnology and Sustainability, Uferstraße 53, Straubing 94315, Germany

## Abstract

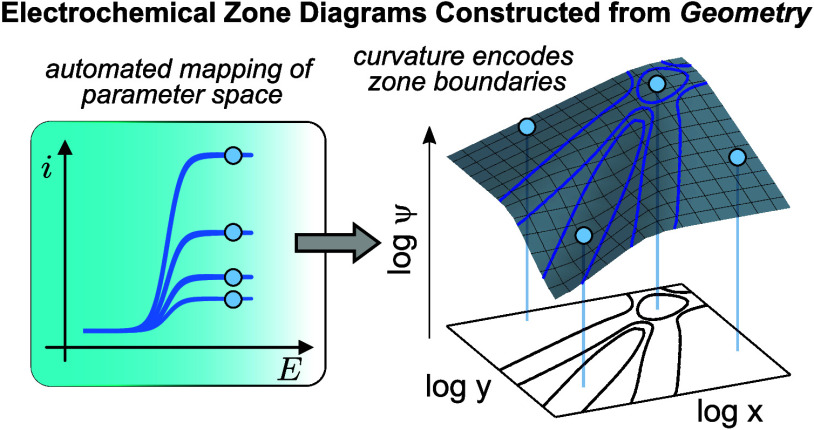

Electrochemical systems with increasing complexity are gaining importance in catalytic energy conversion applications. Due to the interplay between transport phenomena and chemical kinetics, predicting optimization is a challenge, with numerous parameters controlling the overall performance. Zone diagrams provide a way to identify specific kinetic regimes and track how variations in the governing parameters translate the system between either adverse or optimal kinetic states. However, the current procedures for constructing zone diagrams are restricted to simplified systems with a minimal number of governing parameters. We present a computationally based method that maps the entire parameter space of multidimensional electrochemical systems and automatically identifies kinetic regimes. Once the current output over a discrete set of parameters is interpreted as a geometric surface, its geometry encodes all of the information needed to construct a zone diagram. Zone boundaries and limiting zones are defined by curved and flat regions, respectively. This geometric framework enables a systematic exploration of the parameter space, which is not readily accessible by analytical or direct numerical methods. This will become increasingly valuable for the rational design of electrochemical systems with intrinsically high complexity.

## Introduction

Electrochemistry is interfacial. This inevitably couples heterogeneous electron transfer to mass transport and possibly homogeneous reactions in a complex web of physics and chemistry. Disentangling each coupled process is a prerequisite to extracting useful information, such as the mechanism and intrinsic rate constants, from electrochemical experiments. Furthermore, identifying distinct kinetic regimes (encompassing both mass transport and chemical reactions) is essential for the design of important catalytic technologies that mediate the activation of small molecules for energy conversion,^1^ for example, renewable electrosynthesis^2,3^ and photoelectrochemical water splitting.^4^

Zone diagrams provide a solution; these are visual maps that show how transitioning between disparate kinetic regimes depends on operational and intrinsic parameters (typically consisting of rate constants, bulk concentrations, diffusion coefficients, catalyst layer thickness, etc.), ultimately allowing for rational design. To that end, kinetic zone diagrams have long served as a roadmap to guide theory and experiment in analyzing and optimizing any given electrochemical system.^5−7^

Experimental investigations into specific electrochemical systems have proliferated; however, complete descriptions of all limiting kinetic behaviors for individual mechanisms, arranged visually in a zone diagram, remain relatively scarce. A few recent examples include PCET catalysis,^8,9^ gas-diffusion electrodes,^10^ and electron transfer at metal-oxide surfaces.^11^

The common methods for constructing zone diagrams involve solving systems of coupled ordinary or partial differential equations with analytical methods. The frequent presence of nonlinear reaction terms generally does not permit exact solutions. Limiting kinetic behaviors are then obtained by seeking *asymptotic* solutions. This often requires the use of advanced methods for solving differential equations, such as integral transforms (most notably the namesakes of Laplace and Fourier),^12,13^ possibly special functions,^14^ or in the unruliest of cases, perturbation theory.^15,16^

Electrochemical systems featuring a multitude of parallel processes, such as mass transport, electron transfer, and catalytic reactions, are inherently complex because the overall kinetic state of the system depends on a large number of parameters. Even with careful analysis, it will be difficult to manually probe the entire parameter space of large dimensional electrochemical systems using an analytical approach. Additionally, the onset of nonlinear kinetics as a function of one or more parameters is typically abrupt,^17^ creating zones that occupy small regions of the parameter space, and, as a result, optimal kinetic regimes may be missed.

Furthermore, empirical investigations are able to interrogate only a relatively narrow set of all possible kinetic behaviors simply because of the large number of experiments required. Direct numerical simulations, when implemented with dimensional parameters, are of the same flavor. Many individual “parameter sweeps” must be run to determine trends, which themselves do not uniquely identify distinct zones or limiting kinetic behavior. As a result, these methods will also not readily detect certain zones, particularly those associated with a narrow range of parameter values.

To create zone diagrams for electrochemical systems with high complexity, we have developed a computational-based technique that automatically identifies kinetic regimes and generates bespoke zone diagrams for any desired mechanism, even those that would be impractical for the methods mentioned above.

**Figure 1:**
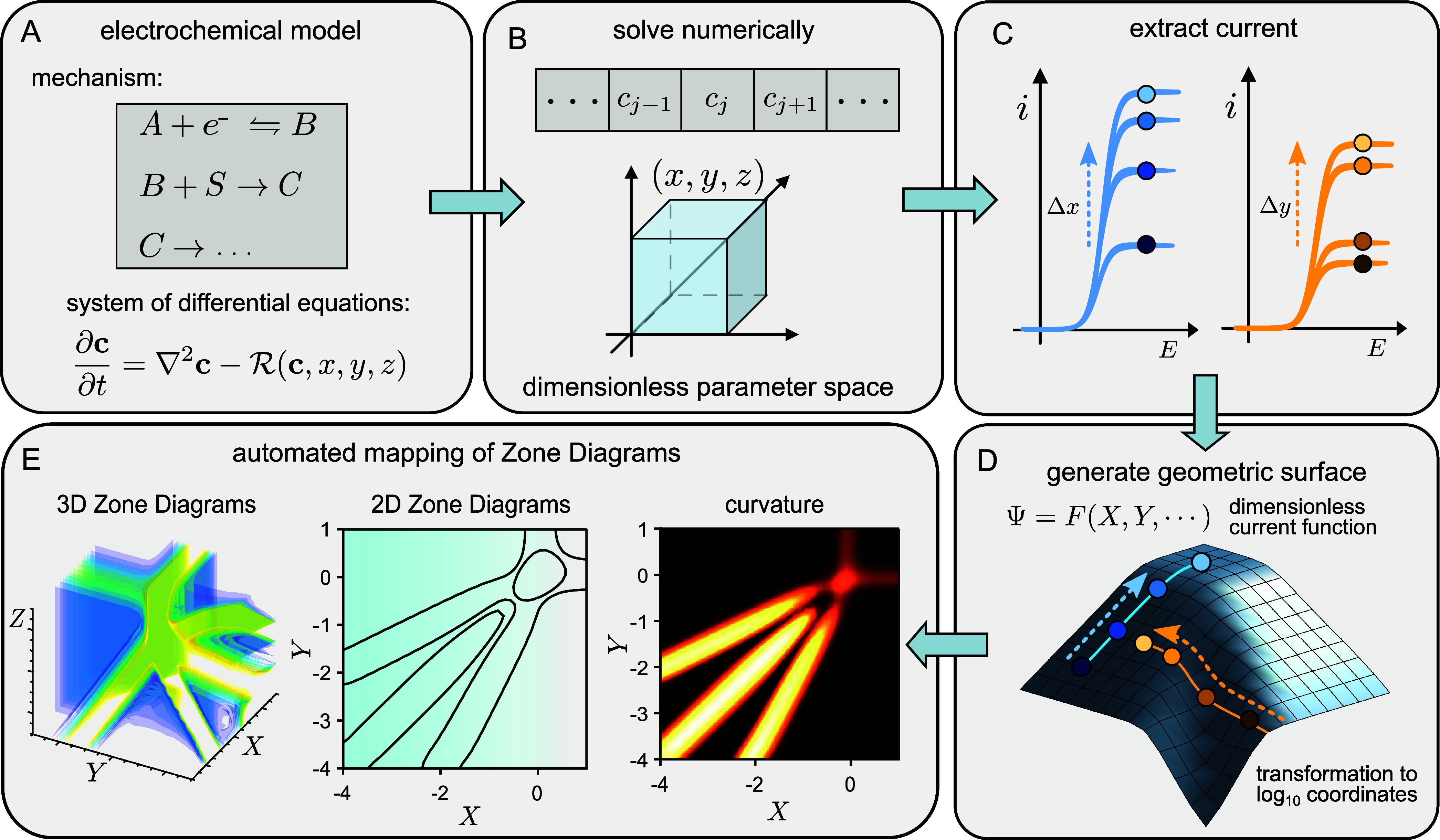
General outline of a geometric approach to zone diagram construction. (A) Any desired electrochemical system with a given mechanism can be represented by a continuum model comprised of a unique set of differential equations. (B) The system is solved using numerical simulations over a discrete set of values for the dimensionless governing parameters (*x*, *y*, and *z*), called the *parameter space*. These are composed of ratios or products of the operations and intrinsic parameters such as rate constants, bulk concentrations, diffusion coefficients, etc. (C) This results in a corresponding set of values for the current, which can be extracted from simulated electrochemical data. (D) The dimensionless *current function* quantifies how the current depends on the governing parameters. Following a transformation to logarithmic coordinates, the dimensionless current function Ψ = *F*(*X*, *Y*, ···) is represented as a geometric surface, with the dimensionless governing parameters expressed in logarithmic coordinates as *X* ≔ log *x*, *Y* ≔ log *y*, and *Z* ≔ log *z*, and Ψ is the logarithm of the dimensionless current. (E) Computing the curvature of this abstract surface reveals zone boundaries. Automation of this procedure allows for the facile construction of zone diagrams in either two or more dimensions for any electrochemical system and mechanism.

Herein, we show that the current response of any electrochemical system is a multivariable function whose graph can be interpreted as a geometric surface, parametrized by each of the governing dimensionless parameters. Once a model is nondimensionalized and implemented numerically (for example, in popular software such as COMSOL^18^ and DigiElch^19^), this surface can be readily generated (Figure 1A–D). The key insight of this approach is that all of the information needed to construct a zone diagram is encoded in the geometry of such an abstract surface. We show how locally curved regions directly correspond to zone boundaries. Thus, we convert the entire process of zone diagram construction into a relatively simple geometric problem of finding curvature (Figure 1E).

While this method is in principle amenable to any electrochemical system, we focus on constructing zone diagrams with examples from molecular catalysis of electrochemical reactions under either homogeneous or heterogeneous conditions. This is advantageous because catalytic mechanisms usually permit steady-state solutions (otherwise called *pure kinetic conditions*),^12^ allowing us to calibrate the geometric interpretation of zone diagrams presented here. Since we are dealing with catalysis, we will consider the main observable to be the steady-state plateau current. However, the same methodology can be applied to any well-defined observable in the experiment, such as the peak current, peak potential, or half-wave potential.

It is important to note that this is not meant to replace the aforementioned analytical techniques, which ideally yield approximate (or, in rare cases, exact) closed-form expressions for the current. The value of such an analysis cannot be overstated. The expression for the current response in each limiting kinetic behavior tells us exactly how the current depends on each intrinsic and operational parameter, which is consequently a key step in predicting rational design. However, we hope that the geometry-based numerical method presented here serves as a foothold for the field to rapidly diagnose the growing complexity of new electrochemical systems.

Although zone diagrams offer essential predictions for guiding kinetic optimization, their meaning is often not well understood, and as a result, they are rarely utilized. To remedy this and provide a primer for the field, we start with a brief analysis of zone diagrams: their basic components, the information they contain, and the existing techniques used to construct them. In the process, we will demonstrate the importance of dimensionless governing parameters and discuss what they are, how to determine their total number in any given system, and what insights they hold.

We will then transition to a geometric point of view, delineating step by step how to construct a two-dimensional (2D) zone diagram using differential geometry. First, the logarithm of the current function is interpreted as a geometric surface. After accomplishing this, the key step in constructing a zone diagram distills down to identifying where the surface is curved. We show that flat and curved regions represent limiting kinetic zones and transition zones, respectively. Next, several catalytic mechanisms are examined as case studies, where we present the automated generation of their respective zone diagrams derived from geometric considerations. Finally, the generality of this approach for higher dimensions is discussed, showing its applicability first to three-dimensional zone diagrams and then to any number of dimensions.

## Results and Discussion

### Anatomy of a Zone Diagram

**Figure 2:**
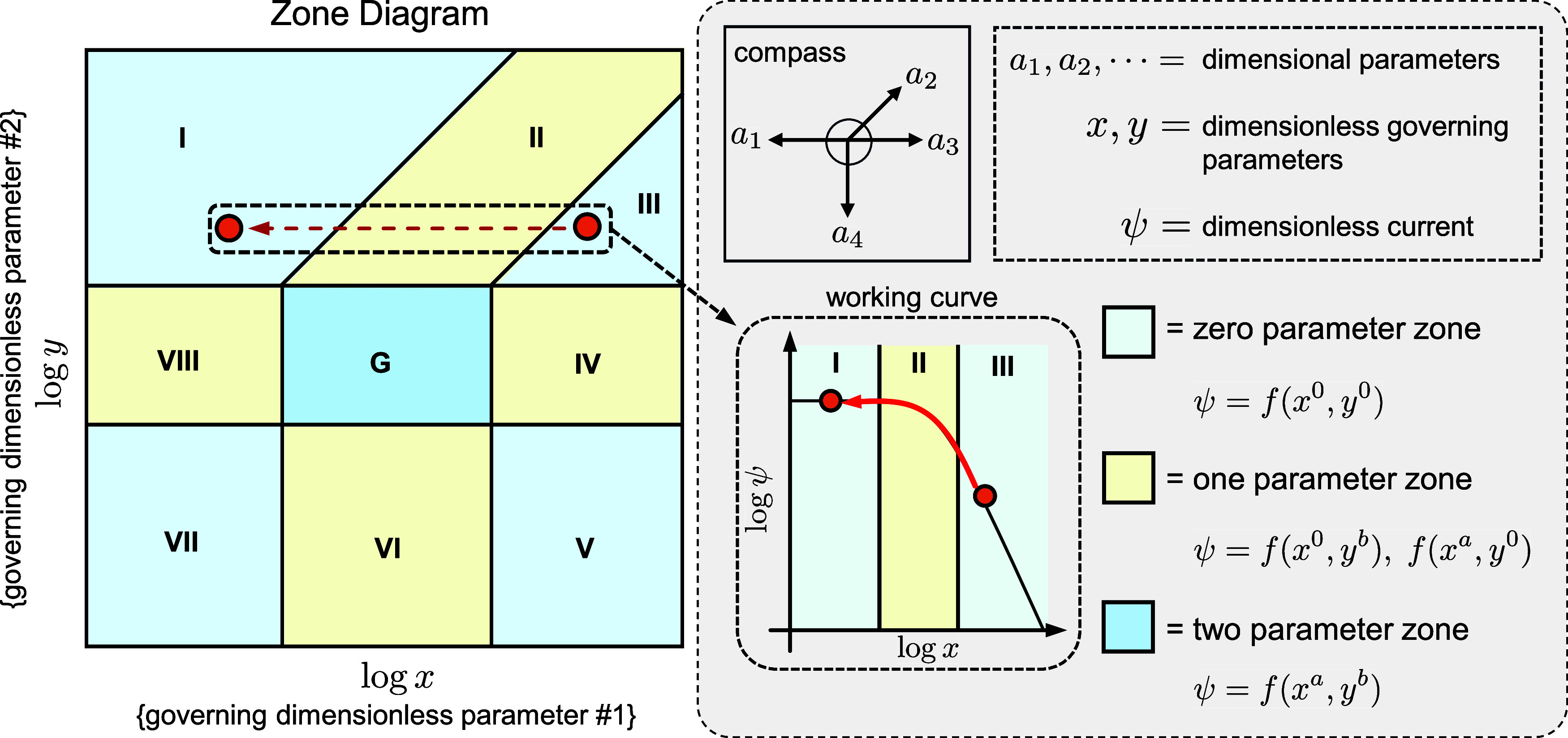
Anatomy of a zone diagram. A general zone diagram is displayed to highlight the important features. Each zone corresponds to a specific kinetic behavior and is labeled with a desired nomenclature (typically numerals or short abbreviations). The vertical and horizontal axes are plotted as the common logarithm of the two dimensionless governing parameters (labeled here as *x* and *y*). These are functions of the dimensional intrinsic and operational parameters *a*_1_, *a*_2_, *a*_3_, ···. The direction in which each dimensional parameter translates the system in the zone diagram is indicated by vectors in a compass rose. Visualizing the effect of a specific transition (changing one or more dimensional parameters) on the current output is possible if one of the dimensionless parameters is held constant. An example is displayed by the red dotted arrow showing the translation from zone III to I as *x* is varied. A working curve results where the dimensionless current log ψ is plotted versus log *x*. Such a “slice” is a one-dimensional projection of the full two-dimensional zone diagram. Transition zones are classified as two-parameter (G) or one-parameter (II, IV, VI, and VIII) and inhabit regions between limiting kinetic behaviors. Here, the approximate current expression depends on one or more governing dimensionless parameters. Zero-parameter zones, where the current is independent of all dimensionless governing parameters, are designated by I, II, V, and VII.

For a particular electrochemical system (defined by the mechanism, mode(s) of mass transport, electrode geometry, and boundary conditions), a zone diagram is a map that tracks characteristic kinetic behaviors (Figure 2). A point on the map represents the state of the system defined by a set of values for the operational and intrinsic parameters, such as bulk concentrations and intrinsic rate constants. Unique kinetic regimes are identified by two-dimensional bordered regions called zones^20^ or sometimes cases.^21−23^

Changing these parameters will shift the current response from one kinetic behavior to another, translating the system’s state in a particular direction between different zones. Extending the map analogy, specific changes in each parameter are the cardinal and intercardinal directions (the points on a compass) for a zone diagram, which is why they are typically displayed alongside zone diagrams as a compass rose (Figure 2). For example, increasing the bulk concentration of substrate may trigger a change in the rate-limiting step of the reaction, or modifying the thickness of the catalyst layer in a gas-diffusion electrode may modulate the impact of mass transport on the current, each signal crossing into a new zone.

Zones that represent limiting kinetic behavior often have an approximate analytical expression that shows how the current response depends on operational and intrinsic parameters. These expressions can be used to diagnose kinetic behavior (determining one’s location on the map). Once the state of the system is located on the zone diagram, its primary utility is precisely predicting which parameters to adjust and by how much to optimize the system. In other words, achieving a desired kinetic behavior amounts to plotting a course through the *parameter space* of an electrochemical system guided by the corresponding zone diagram.

Zones can be classified as either zero-, one-, or two-parameter zones (Figure 2).^24^ A zero-parameter zone occurs when the asymptotic approximation to the current response formally does not depend on any dimensionless governing parameters (these are composed of products or ratios of the dimensional operational and intrinsic parameters and will be discussed further in the next section); this is synonymous with limiting kinetic behavior. For example, a zero-parameter zone may reflect the control of the current by a particular rate-limiting chemical step or mode of mass transport. Sometimes, it may not be apparent that the current in a zero-parameter zone is independent of the dimensionless governing parameters. However, the zero-parameter current can always be appropriately rescaled to reveal this invariance.

In contrast, transition zones fall into the one- and two-parameter categories, where the asymptotic approximation to the current expression formally depends on one or two governing dimensionless parameters, respectively.^25,26^ These transition zones always lie between two different zero-parameter zones, creating pathways between distinct limiting kinetic regimes. Traversing these paths between zones occurs with any change in the governing dimensionless parameters.

### Governing Dimensionless Parameters: What Are They and How Are They Found?

Governing dimensionless parameters are central to zone diagram construction. Each one will form a single axis on a logarithmic scale of the resulting diagram. Consequently, the total number of governing dimensionless parameters determines the dimensionality of the corresponding zone diagram. For example, if a particular electrochemical system has only one dimensionless parameter, the resulting zone diagram is one-dimensional and simply represented by a line. Historically, these one-dimensional zone diagrams are called “working curves” because the current function (or some other observable) is visualized straightforwardly as a graph with the single governing parameter on the horizontal axis and the value of the current on the vertical axis.

Perhaps the most well-known working curve is that of Nicholson for single electron transfer to a freely diffusing species probed by cyclic voltammetry (CV).^27^ It shows the progression from electrochemical reversibility to irreversibility by relating the peak potential separation to the scan rate. The single dimensionless parameter here is the ratio of the rate of interfacial electron transfer to the rate of diffusion. Analogously, if there are two dimensionless governing parameters controlling the current response, a two-dimensional zone diagram will result, and so on for higher dimensions.

Dimensionless parameters, or dimensionless groups as they are sometimes called, are defined by a number of dimensional parameters (rate constants, concentrations, diffusion coefficients, etc.) grouped as products and ratios in such a way that their units (more accurately, their dimensions) cancel out, leaving a dimensionless number. *Importantly, the current response is ultimately controlled by these dimensionless groups rather than individual dimensional parameters*.

For example, in the classic CV response of a simple homogeneous catalytic reaction, the rate of catalysis and the scan rate both affect the shape of the CV in a parallel manner. Under negligible substrate depletion, increasing the catalytic rate constant or decreasing the scan rate both push the system in the same direction toward pure kinetic conditions, characterized by a sigmoidal CV at pseudosteady state.^12,30,31^ In this case, the current–potential response does not depend on the catalytic rate constant or the scan rate individually, but as a ratio, contained in a dimensionless group λ = *RTk*_cat_/*F*ν (*k*_cat_ is the pseudo-first-order rate constant, ν is the scan rate, *R* is the gas constant, and *T* is the temperature).

In more technical terms, if we consider a vector space, with individual dimensional parameters represented as vectors, pointing in the direction in which increasing their value shifts the system within the zone diagram, dimensional parameters that have a complementary effect on the current *span the same subspace* (see, for example, the parameters *a*_1_ and *a*_3_ in the compass rose in Figure 2). Consequently, parameters that lie on the same span also appear together in the same dimensionless governing parameter as either a ratio or product.

This leads to the conclusion that the state of any electrochemical system can be uniquely defined only after it is nondimensionalized and the dimensionless governing parameters are identified. Continuum models implemented with dimensional variables will inherently be unable to map all possible kinetic zones, as multiple sets of dimensional parameters can correspond to the same state of the system.

Returning to the catalytic CV example, collecting the dimensional variables into dimensionless groups effectively reduced the total number of input parameters in the problem from two originally (*k*_cat_ and ν) to only a single one, λ. Making an electrochemical system dimensionless, expressed in terms of dimensionless governing parameters, greatly simplifies the mathematical form of the problem and reveals latent physical insights, in this case, the relationship between the rate of catalysis and the scan rate.

The total number of governing dimensionless parameters in an electrochemical system is found by dimensional analysis simply by observing the fundamental dimensions contained in each parameter or independent variable.^32^ Although not often discussed in the electrochemical literature, *Buckingham*’*s Π theorem* formalizes this procedure.^33^ Consider a physically meaningful equation *g* that relates the current *i* to *n-*dimensional parameters *a*_1_, *a*_2_, ···, *a*_*n*_, expressed as
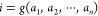
The theorem states the total number of *dimensionless* governing parameters  is given by
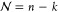
where *n* represents the total number of physical variables (dimensional parameters) and *k* denotes the total number of fundamental dimensions in the system. These fundamental dimensions may include, for example, any of the base quantities: length, time, mass, electric current, amount of substance (moles), temperature, or luminous intensity. This leaves a dimensionless equation *f* of the form
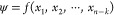
where *x*_1_, *x*_2_, ···, *x*_*n*–*k*_ are the governing dimensionless parameters and ψ is the dimensionless current. Buckingham’s Π theorem tells us about the total number of independent dimensionless groups to expect but not what form they take. As a result, *the dimensionless governing parameters for a given electrochemical system are not unique*. There will be multiple ways to nondimensionalize an electrochemical problem, and finding the most physically relevant set of governing parameters is a challenge. This could, in principle, be aided by the automated computational-based approach presented here, which allows for the rapid screening of different sets of dimensionless parameters.

### Zone Boundaries the Traditional Way

**Figure 3:**
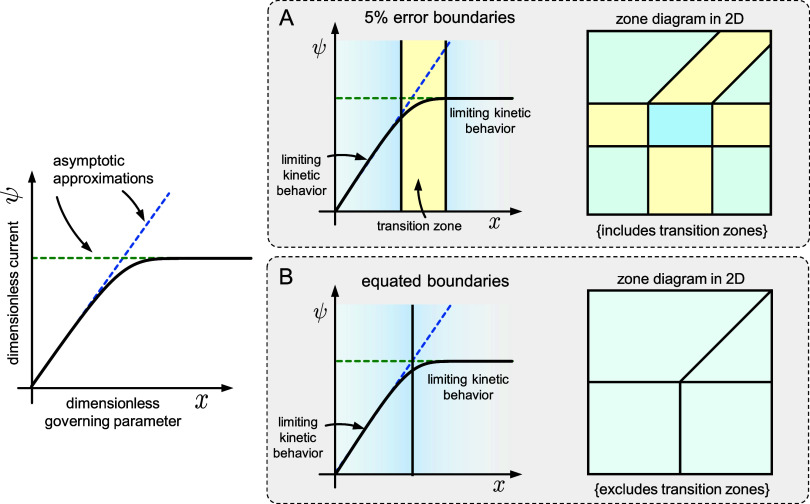
Example working curves (one-dimensional zone diagrams) highlighting definitions of zone boundaries based on either (A) equated boundaries^21,28^ or (B) percent error boundaries.^10,29^ The current function (bold black line) and two limiting asymptotic approximations to the current function (dotted green and blue lines) are shown on the working curve on the left. Each definition is applied, resulting in different placements of the zone boundaries (vertical lines). Examples of 2D zone diagrams based on these definitions are shown on the right.

The important step in constructing a zone diagram is to determine where zone boundaries lie in the parameter space (Figure 3). Finding these first requires modeling the electrochemical system with a set of differential equations and boundary conditions. Traditionally, the next step is to examine the limiting kinetic behavior by setting the governing dimensionless parameters to either very large or very small values. Looking at each limiting kinetic regime allows for simplifying approximations and eventually solving this system of equations using analytical methods. This nets a set of *asymptotic approximations* for the current response, each of which describes a particular limiting kinetic regime. Zones are defined by the corresponding regions in the parameter space, where these asymptotic solutions are valid. With this understanding, zone boundaries are traditionally located by one of two methods.

In the first method (Figure 3A), the asymptotic solutions for the current are simply equated, yielding an analytic algebraic condition for the location of the zone boundary (e.g., *y* = *f*(*x*), where *x* and *y* are the two dimensionless governing parameters).^21,34^ This identifies where the two asymptotic approximations coincide. While an algebraic expression for the zone boundary is quite useful, this method omits transition zones (it is not possible to tell if a zone exists between where different asymptotic approximations are valid).

The second means of finding zone boundaries commonly employed (Figure 3B) is to determine where each asymptotic approximation for the current deviates from its actual value by more than a specified tolerance (typically taken as five to ten percent relative error).^10,24,29^ Here, the actual value of the current is obtained from numerical simulations. This method will also identify transition zones (one or two parameters) on the 2D zone diagram. Mathematical definitions for these two types of zone boundaries are given in the Supporting Information (eqs S1 and S2; Section 1).

### Zone Diagrams and Geometry

Now, we will transition to analyzing the parameter space for a general electrochemical system using geometry. Staying within the realm of two dimensions for the moment, the dimensionless current, denoted by ψ, depends on only two dimensionless governing parameters, which, as before, we will call *x* and *y*. These are strictly positive since the intrinsic and operational parameters, including, for example, rate constants, concentrations, diffusion coefficients, etc., are always positive numbers. The dimensionless parameters are represented more compactly by a two-dimensional vector, **x** = (*x*,*y*).

We are now set to define the current as a function more precisely: let  and , then the *current function* for **x** ∈ *D* is defined as
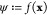
The domain of the current function *D* consists of all possible values for the governing dimensionless parameters and defines the *parameter space* of the system. Fundamentally, zone diagrams illustrate how the behavior of the current changes as this space is traversed (in this case, the positive quadrant of the (*x*, *y*) plane).

**Figure 4:**
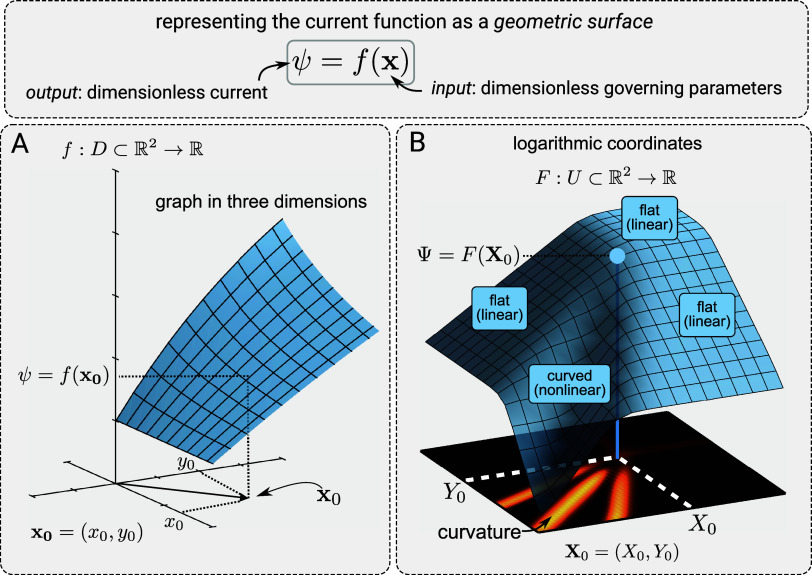
Representing the current function as a geometric surface (A) as a graph plotted in three dimensions. The domain  is the set of all ordered pairs of positive real numbers, i.e., all possible values for the governing dimensionless parameters. The input values **x** = (*x*, *y*) sit on the positive portion of the Cartesian plane. Points on the surface are determined by the height of the graph above the plane, i.e., the value of the dimensionless current ψ at **x**. (B) Transformation to logarithmic coordinates (eq 3). The current function Ψ = *F*(**X**) is defined over the domain , where **X** = (*X*, *Y*) ∈ *U*. Flat regions on the surface correspond to **X** values, where the current function is linear and represents limiting kinetic regimes. Curved regions correspond to **X** values where the current function is nonlinear and signify transition zones. A heat map showing the magnitude of the curvature is displayed below the surface. Curved regions are orange, while flat regions are shown in black.

A deep connection to geometry is not far away: since the current is a function of two variables, plotting its graph results in a two-dimensional surface (Figure 4A) sitting in three-dimensional space. The input is a point on the (*x*,*y*) plane, and the current output ψ represents the height of the graph above the plane (current is defined here as positive for convenience). Another way of representing surfaces as geometric objects, which will be increasingly valuable later, is through parametrization. One simple parametrization representing the same surface is
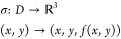
where points on the surface are ordered triplets of the form (*x*, *y*, *f*(*x*, *y*)). More specifically, the function σ is a map that takes points in a region of the plane into three dimensions. Such a transformation is also called a *parametrized surface* (Figure S1). To visualize this, imagine laying a piece of paper flat on a table. Then, it is lifted above the table by picking it up by its edges, bringing it into the third dimension, while at the same time, for example, moving two of its corners closer together. The paper will pucker into a smooth 2D surface sitting in a three-dimensional space.

We will now explore how our geometric picture leads naturally to what defines a zone. To do this, two facts are required. The first of which is limiting forms of the current function can always be written as a *power lawmonomial*. Since we are interested in representing limiting kinetic behavior on the zone diagram, this requires examining the current function in the limits when the dimensionless governing parameters are either very large or very small. In these limits, the current function takes the form of a single term that is a product of each dimensionless parameter raised to a power, otherwise called a power law monomial; hence
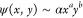
1as (*x*, *y*) → (0, 0), (0, ∞), (∞, 0), or (∞, ∞), where α is an arbitrary constant. The symbol “∼” is read as “is asymptotic to.” Formal definitions and validation for eq 1 can be found in the Supporting Information (Section 2, p. S4).

The next step is motivated by the fact that the coordinates of a zone diagram are expressed as the logarithm of each dimensionless parameter. Following this convention, the limiting power law monomial approximation for the current function, after taking its logarithm, becomes conveniently a linear expression (or, more accurately, affine, since a constant term comes along for the ride). Applying the result above, we find
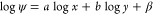
2where β is a second arbitrary constant. At this point, some notational housekeeping is in order. To express the current function on a logarithmic scale, we introduce the following change in variables
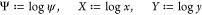
3As before, we write the dimensionless parameters as a vector, **X** = (*X*, *Y*). After this coordinate transformation, there will be a new function *F*, that describes how the logarithm of the current depends on the new input **X**
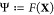
4Now, *X* and *Y* can be positive or negative. Returning to eq 2, the substitution of the new variables yields
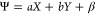
5which approximates the current function for limiting kinetic behaviors now on a log scale. This expression is indeed linear, and when plotted as a graph, it represents the equation for a plane in three dimensions.

### Defining Zones Based on Geometry

Putting all of this together leads neatly to a *geometric definition of a zone*. Limiting kinetic behaviors in a zone diagram are regions of the parameter space where the current function (on a logarithmic scale) is locally linear, i.e., *where its corresponding surface is a flat plane* (Figure 4B). Conversely, this also suggests that the remaining regions of space where the current function is nonlinear, i.e., *where its corresponding surface is curved*, denote transition zones, which connect one zone of limiting kinetic behavior to another (Figure 4B). In the following sections, we will show that once regions are identified as either locally linear or nonlinear or equivalently as either flat or curved, placing boundaries on the zone diagrams follows straightforwardly.

### Quadratic Approximation

Although linear and nonlinear, flat and curved are conceptually the same when viewed under the lens of geometry, before discussing curvature, we will first show how to use the concept of local linearization to construct a zone diagram. The current function, Ψ = *F*(**X**), can be approximated near a point, **X**_0_ = (*X*_0_, *Y*_0_), using the quadratic approximation, given by
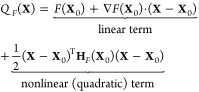
In the first term, *F*(**X**_0_) is a constant, and ∇*F*(**X**_0_) is the gradient of *F* evaluated at **X**_0_, defined as ∇*F* = (∂_*X*_*F*, ∂_*Y*_*F*). The second term includes **H***_F_*(**X**_0_), called the Hessian matrix, also evaluated at **X**_0_. This contains the partial and mixed partial second derivatives of the function *F*, defined as
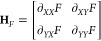
The quadratic approximation is useful here because it simplifies the current function into two parts: a linear term (including the constant) and a quadratically nonlinear term. If the quadratic part is vanishingly small near some input **X**_0_, then the current function is locally linear (the surface is locally flat). Since we showed before that local linearity corresponds to limiting kinetic behavior, a limiting zone must be present in this vicinity of the parameter space.

Conversely, if the quadratic term is nonzero, then the current function is locally nonlinear (the surface is locally curved), signaling a transition zone around that point. The quadratic term approaches zero if the Hessian matrix, evaluated at **X**_0_, tends toward the null matrix (where all of the entries are zero). Computationally, the “magnitude” of **H**_*F*_ at an input can be described by its norm, ∥**H**_*F*_(**X**_0_)∥. In a sense, this indicates how close it is to the null matrix. Thus, to place zones on the desired zone diagram, we look point-by-point over a discrete domain of the dimensionless governing parameters, classifying regions as either locally linear or nonlinear by calculating the norm of the current function’s Hessian matrix at each point.

### Curvature Encodes Zone Boundaries

The process of identifying zone boundaries has been reduced to a search for areas that are flat on the geometric surface obtained from the log of the current function, which is a representation of the parameter space of a general electrochemical system. Decoding the geometric structure of this abstract surface involves defining and calculating the curvature. Here, we provide a brief description of the concept of a curved surface and how curvature can be quantified; more detailed definitions and computational methods for calculating curvature can be found in the Supporting Information (Section 3, p. S5).

**Figure 5:**
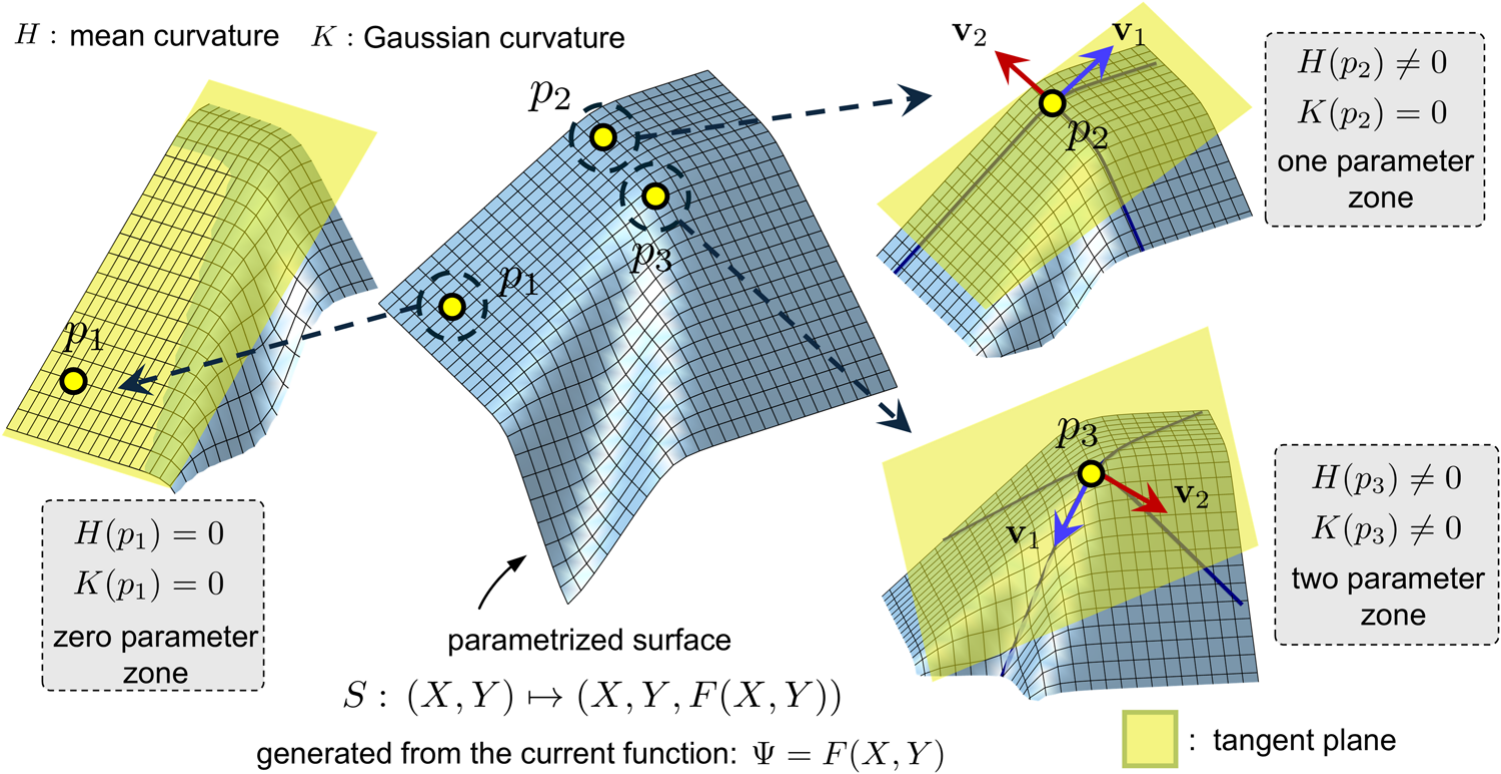
Classification of zones based on the mean and Gaussian curvature. The parametrized surface *S* is displayed as the graph of the current function Ψ. The points *p*_1_, *p*_2_, and *p*_3_ on *S* correspond to points in the parameter space. These are classified as being located in either zero-, one-, or two-parameter zones on the basis of the value of the mean curvature *H*(*p*) and the Gaussian curvature *K*(*p*). The tangent plane to *S* at each point is displayed in yellow. The principal directions, **v**_1_ and **v**_2_, are indicated with red and blue vector arrows. The plane curves on *S* corresponding to each principal direction are shown as dark blue paths on the surface.

On a two-dimensional surface, at any point *p*, the two directions in which the surface bends away the most (a sharp curve) and the least (a gentle curve) from the ambient space are referred to as the *principal directions*. We will represent these directions with vectors **v**_1_ and **v**_2_ (Figure 5). Each principal direction corresponds to a *principal curvature*, κ_1_ and κ_2_ (the respective values of the curvature in each of these directions). If one were to walk along the surface at constant speed in a line tracing out a path in each of the principal directions, the amount that this path curves away from the starting point *p* would give an intuition for the magnitudes of κ_1_ and κ_2_. Taking the average of the principal curvatures results in what is called the *mean curvature H*
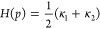
and their product is the *Gaussian curvature K*
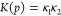
Based on these definitions of curvature, we can develop criteria to discriminate between zero-, one-, and two-parameter zones. In our notation, a point **X** = (*X*, *Y*) in the parameter space maps to the point *p* = (**X**, *F*(**X**)) on the surface, where *F*(**X**) is the value of the current Ψ. To begin, by definition, at points where both the mean and Gaussian curvatures are zero or approaching zero (see *p*_1_ in Figure 5), the surface is flat, signifying a zero-parameter zone (which refers to a limiting kinetic regime, as discussed above). Specifically, if *H*(*p*) = 0, this tells us that the corresponding point in parameter space **X** lies in a zero-parameter zone.

In contrast, if *H*(*p*) ≠ 0, **X** lies in a transition zone (see *p*_2_ and *p*_3_ in Figure 5), recall that transition zones are defined as regions of the parameter space where the current formally depends on exactly one or two dimensionless governing parameters, corresponding to the designation one- or two-parameter zones, respectively. In fact, for the purpose of zone diagram construction, the mean curvature conveys essentially the same information as ∥**H**_*F*_(**X**)∥.

More interestingly, Gaussian curvature contains more nuanced information about the zone diagram: it discriminates between different transition zones, allowing us to uniquely identify whether a point in the parameter space lies in either a one-parameter zone or a two-parameter zone. Again, by definition, the current function in a one-parameter zone formally depends on only one dimensionless parameter (i.e., the current is constant with respect to either *X* or *Y*). Translating this into geometric meaning, for a point on the surface corresponding to a one-parameter zone in the parameter space, there will be one direction which, if followed, will not curve away from this starting point.

Consider, for example, *p*_2_ in Figure 5. Walking along the principal direction of **v**_2_ in the vicinity of *p*_2_ would be like walking on a flat plane; one would not experience an incline or decline. Alternatively, walking along the principal direction **v**_1_ does result in curvature. At *p*_2_, one of the principal curvatures is zero and one is nonzero (κ_1_ = 0, κ_2_ ≠ 0), giving *H*(*p*_2_) ≠ 0, and *K*(*p*_2_) = 0. Thus, points on the surface that correspond to a one-parameter zone can be uniquely identified as having a nonzero mean curvature but zero Gaussian curvature.

In fact, this suggests that there is a more fundamental way to identify a zone based on intrinsic geometry. Near the point *p*_2_ in our example, the surface behaves locally like a *cylinder*; it is curved in only one direction and flat perpendicular to that direction. We can actually define one-parameter zones in this way: in the vicinity of a one-parameter zone in the parameter space, the corresponding surface locally has the topology of a cylinder.

Conversely, in a two-parameter zone, the current function depends on both dimensionless governing parameters, so the surface will curve at least in two different directions (the current function is nonconstant with respect to both *X* and *Y*). In this case, for a point on the surface corresponding to a two-parameter zone (consider *p*_3_ in Figure 5), both principal curvatures are nonzero. Therefore, two-parameter zones can be uniquely identified by the criterion that both the mean and Gaussian curvatures are nonzero. Topologically, two-parameter zones are defined as regions of the parameter space where the corresponding vicinity on the surface locally resembles a *sphere*. To summarize, by computing the mean and Gaussian curvature at points on the surface derived from the current function, we can delineate the location in the parameter space of all zero-, one-, and two-parameter zones based on the conditions outlined in Figure 5.

### Case Studies

We now proceed to apply this geometric framework to several known mechanisms from molecular catalysis of electrochemical reactions, which have corresponding zone diagrams in the literature. This will allow us to calibrate the method using well-established models as reference points. Second, we wish to provide step-by-step examples demonstrating how the concepts of geometry and curvature, outlined above, combined with numerical methods for simulating electrochemical systems, can efficiently and rapidly construct zone diagrams. The selected mechanisms span heterogeneous and homogeneous conditions and represent a range of complexity based on the number and reversibility of the individual chemical steps. The diversity present in these examples highlights how the computational nature of this approach lends itself well to automation, particularly across disparate electrochemical systems.

#### Electrocatalytic Films

Catalysis within porous electrodes or electroactive films is becoming increasingly important for the electrochemical conversion of small molecules,^38^ recognizing the need to effectuate high current densities while circumventing mass transport limitations. Early analytical models of such systems, complete with zone diagrams, appeared for molecular catalysts embedded in redox polymer-modified electrodes,^20,22,35,37,39^ with several more recent analyses by cyclic voltammetry from Costentin and Savéant.^24,36,40^

Two diffusional transport processes are at work: mass transport of the substrate within the film as well as charge transport (electron hopping). Here, we consider a single one-electron catalytic step for simplicity. This can be extended to multielectron reactions, as shown by the application of the theory to metal-oxide films for water oxidation.^41^ Let *P* be the oxidized catalyst, *Q* be the reduced catalyst, *S* be the substrate, and *k*_1_ be the second-order catalytic rate constant. The overall reaction scheme is
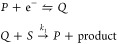
As shown previously, the current response is governed by two dimensionless parameters, which, to match the axes of the original zone diagram,^35,36^ we define here as
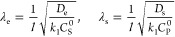
where  is the thickness of the film or catalyst layer, *C*_S_^0^ is the total concentration of substrate, *C*_P_^0^ is the total concentration of catalyst or active sites, *D*_S_ is the diffusion coefficient of the substrate, and *D*_e_ is the charge transport diffusion coefficient. Physically, these describe the competition between charge transport, substrate diffusion, and catalytic reaction. Further details of the model for this mechanism, the governing equations, and a numerical solution for the current response can be found in the Supporting Information (Section 4.1, p. S7).

**Figure 6:**
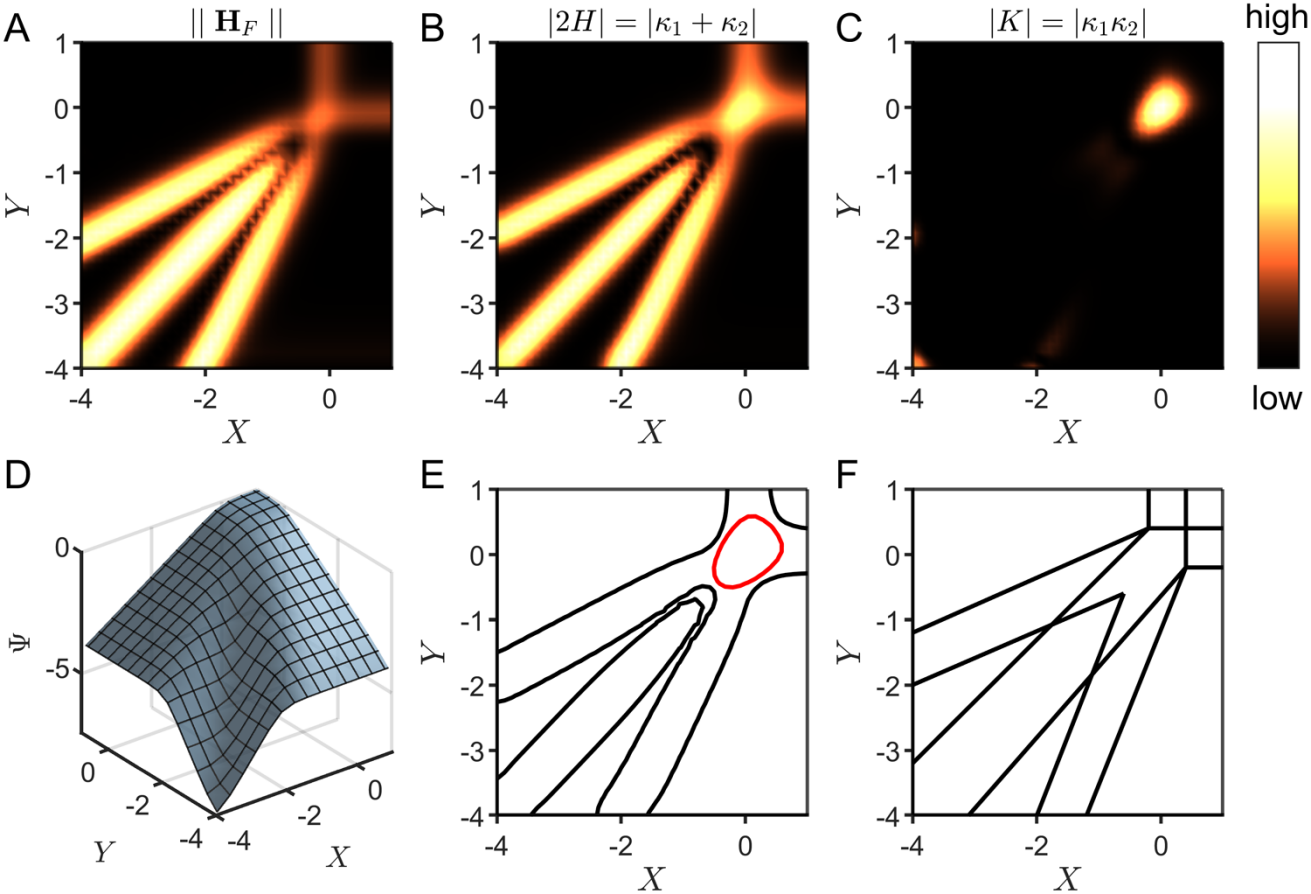
Case study of an electrocatalytic film. Zone diagrams are visualized for this mechanism^35−37^ using both the quadratic approximation and curvature. The norm of the Hessian ∥**H**_*F*_∥ (A) and the absolute values of the mean curvature |2*H*| (B) and Gaussian curvature |*K*| (C) were used to construct heat maps over the two-dimensional parameter space (black regions correspond to low curvature and bright yellow regions correspond to high curvature). The values were obtained from the geometric surface (D) generated for this mechanism using numerical methods to compute the steady-state current. A zone diagram (E) is built from a composite of the mean and Gaussian curvature, where the boundaries are defined as 20% of the maximum mean curvature (zero- and one-parameter zones, black lines) and 20% of the maximum Gaussian curvature (two-parameter zone, red line). (F) The reported zone diagram is displayed for comparison.^36^

Implementing a numerical solution for this system over a discrete set of the two dimensionless governing parameters allows us to generate a geometric surface (Figure 6D) from the current function. Here, the parameter space  is defined by *X* = log λ_e_ and *Y* = log λ_s_. When plotted as heat maps, both the norm of the Hessian ∥**H**_*F*_∥ and the absolute value of the mean curvature |2*H*| and Gaussian curvature |*K*| reveal distinct zones and are displayed in Figure 6A–C. Limiting zero-parameter zones are signaled by dark regions where ∥**H**_*F*_∥ → 0 and |2*H*| → 0, while transition zones are displayed in orange where the curvature is nonzero. Additionally, the Gaussian curvature reveals a central two-parameter zone (Figure 6C, orange region; |*K*| > 0).

By overlaying the mean and Gaussian curvatures, we can construct the zone diagram shown in Figure 6E. Zone boundaries are displayed as a specific contour of the maximum mean or Gaussian curvature, where the two-parameter zone identified by the Gaussian curvature is distinguished with a red boundary. Thus, we provide here for the first time a mathematical definition for zone boundaries based on curvature (see the Supporting Information, eqs S3–S5).

The reported zone diagram for this mechanism, in which zone boundaries are defined by percent error (see Figure 3B and eq S2, Supporting Information), is reproduced in Figure 6F.^36^ By comparison, the construction using a composition of mean and Gaussian curvatures (Figure 6E) effectively reproduces the reported zone diagram, with small variations in the exact location of the zone boundaries. This is to be expected given that the mathematical definitions of the zone boundaries differ (see eqs S1–S5, Supporting Information). For example, near the center of the zone diagram in Figure 6E at **X** ≈ (−0.7, –0.7), a narrow gap appears between the one-parameter and two-parameter zones, which is not present in Figure 6F. The reason for this is a *change in the sign of the curvature*.

Proceeding from the top right corner **X** = (1, 1) to the bottom left corner **X** = (−4, –4) along the middle diagonal (i.e., the line defined by *Y* = *X*) in Figure 6D, it is clear that the graph of the current function switches from concave down to concave up. We know from the model that this sign flip in the curvature coincides with the interference of an additional diffusional process—either charge transport or substrate diffusion—in the kinetic control of the current. Interestingly, this suggests that the magnitude and sign of the curvature are important metrics that provide additional information about the system (*vida infra*).

#### Mediated Enzymatic Catalysis in a Redox Film

Staying within the realm of catalytic films and heterogeneous catalysis, the next case study adds complexity to the catalytic mechanism. Bartlett and Pratt introduced a model and zone diagram for enzymatic catalysis within a polymer-film-modified electrode driven by outer-sphere electron transfer via an immobilized redox-active mediator.^21,42^ The overall sequence of chemical and electrochemical steps is
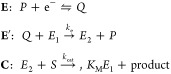
where now *P*/*Q* denotes the oxidized and reduced form of the mediator, *E*_1_/*E*_2_ denotes the oxidized and reduced states of the enzyme at a total concentration given by *C*_E_^0^, and *S* is the substrate, as before. The mediated outer-sphere electron transfer step (**E′**) is modeled as a simple bimolecular reaction with a second-order rate constant *k*_e_, and the reaction between enzyme and substrate (**C**) is described by Michaelis–Menten kinetics (with a catalytic rate constant *k*_cat_ and the Michaelis–Menten constant *K*_M_). Again, two dimensionless parameters govern the current response (more are present in the model, but we restrict ourselves to two dimensions for the moment). These are defined as
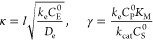
These dimensionless groups also have concrete physical meanings. Briefly, κ describes the ratio of the rate of charge transport within the film to the rate of electron transfer between the mediator and the enzyme, and γ determines the rate-controlling chemical step in the film: either mediated electron transfer or enzymatic turnover.

**Figure 7:**
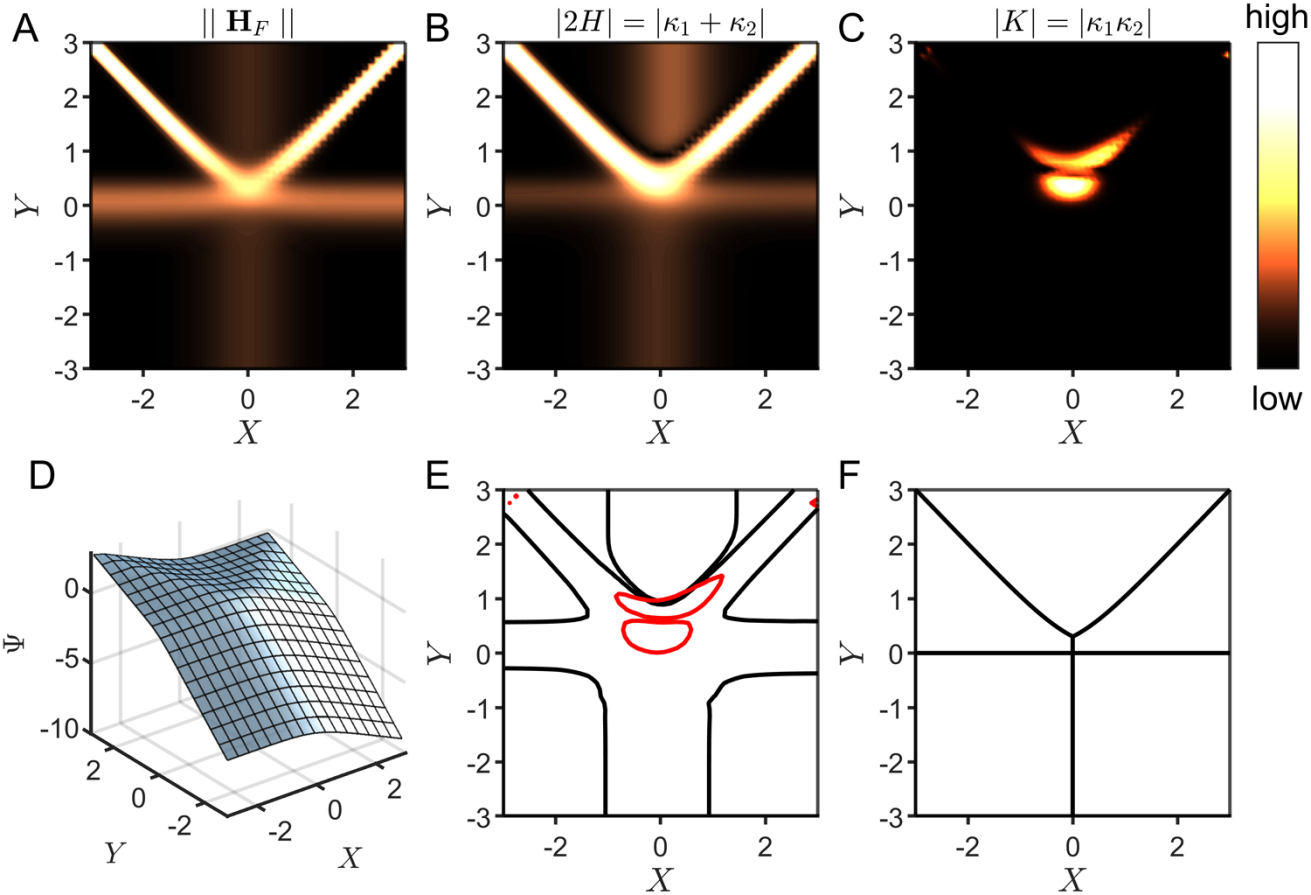
Case study of mediated enzymatic catalysis in a redox film.^21^ Zone diagrams are visualized using both the quadratic approximation and curvature. The norm of the Hessian ∥**H**_*F*_∥ (A) and the absolute values of the mean curvature |2*H*| (B) and Gaussian curvature |*K*| (C) were used to construct heat maps over the two-dimensional parameter space (black regions correspond to low curvature and bright yellow regions correspond to high curvature). The values were obtained from the geometric surface (D) generated for this mechanism using numerical methods to compute the steady-state current. A zone diagram (E) is built from a composite of the mean and Gaussian curvature, where the boundaries are defined as 2% of the maximum mean curvature (zero- and one-parameter zones, black lines) and 20% of the maximum Gaussian curvature (two-parameter zone, red line). (F) The reported zone diagram is displayed for comparison.^21^

Turning to analyzing the parameter space of this system with geometry, we define *X* = log γ and *Y* = log κ. As before, after generating the geometric surface (Figure 7D) from a numerical solution for the current function, zones are identified using ∥**H**_*F*_∥, |2*H*|, and |*K*| (Figure 7A–C). Their composition yields the zone diagram in Figure 7E, with zone boundaries placed on a contour of the curvature values.

In this case, the reported zone diagram (reproduced in Figure 7F)^21^ used equated boundaries (see Figure 3A and eq S1, Supporting Information), where transition zones (one and two parameter) are merged with limiting kinetic behavior (zero parameter) zones. Thus, we see, for the first time, the emergence of the transitional behavior for this system (orange regions, Figure 7A–C), obtained by locating nonzero curvature. Indeed, the zone identified as Case III^21^ is actually split into two zero-parameter zones, separated by a large transitional one-parameter zone (Figure 7E). This raises the interesting possibility that mapping the parameter space using this geometric framework can discover entirely new kinetic behavior (*vide infra*).

#### CO_2_ Reduction by a Homogeneous Molecular Fe(0) Catalyst

We now examine a multistep, multielectron reaction under homogeneous conditions. In the reduction of CO_2_ by molecular catalysts, a class of iron porphyrin complexes display an archetypal mechanism.^43−46^ In-depth kinetic studies of these systems have revealed insights that provide the basis for our general knowledge of this important reaction, including the nature of C–O bond breaking, the role of proton sources, hydrogen bonding, second-coordination-sphere effects, linear free-energy relationships, and factors driving product selectivity. The mechanism is displayed below
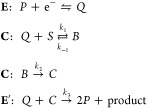


The essential features follow an **ECCE′** mechanism initiated by electrochemical (**E**) generation of the active Fe(0) catalyst *Q*. This is followed by the reversible binding of CO_2_ (**C**) to form the initial CO_2_-bound adduct *B*. Next, the breaking of the C–O bond (**C**) forms an iron carbonyl species *C*. Finally, the cycle is closed by a homogeneous electron transfer step (**E′**), releasing the product (carbon monoxide) and regenerating the oxidized form of the catalyst *P*. Assuming the final homogeneous electron transfer described by *k*_3_ is fast, the two dimensionless governing parameters are composed of ratios of the rate constant for the different chemical steps and determine which is rate-controlling, defined as
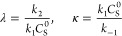


**Figure 8:**
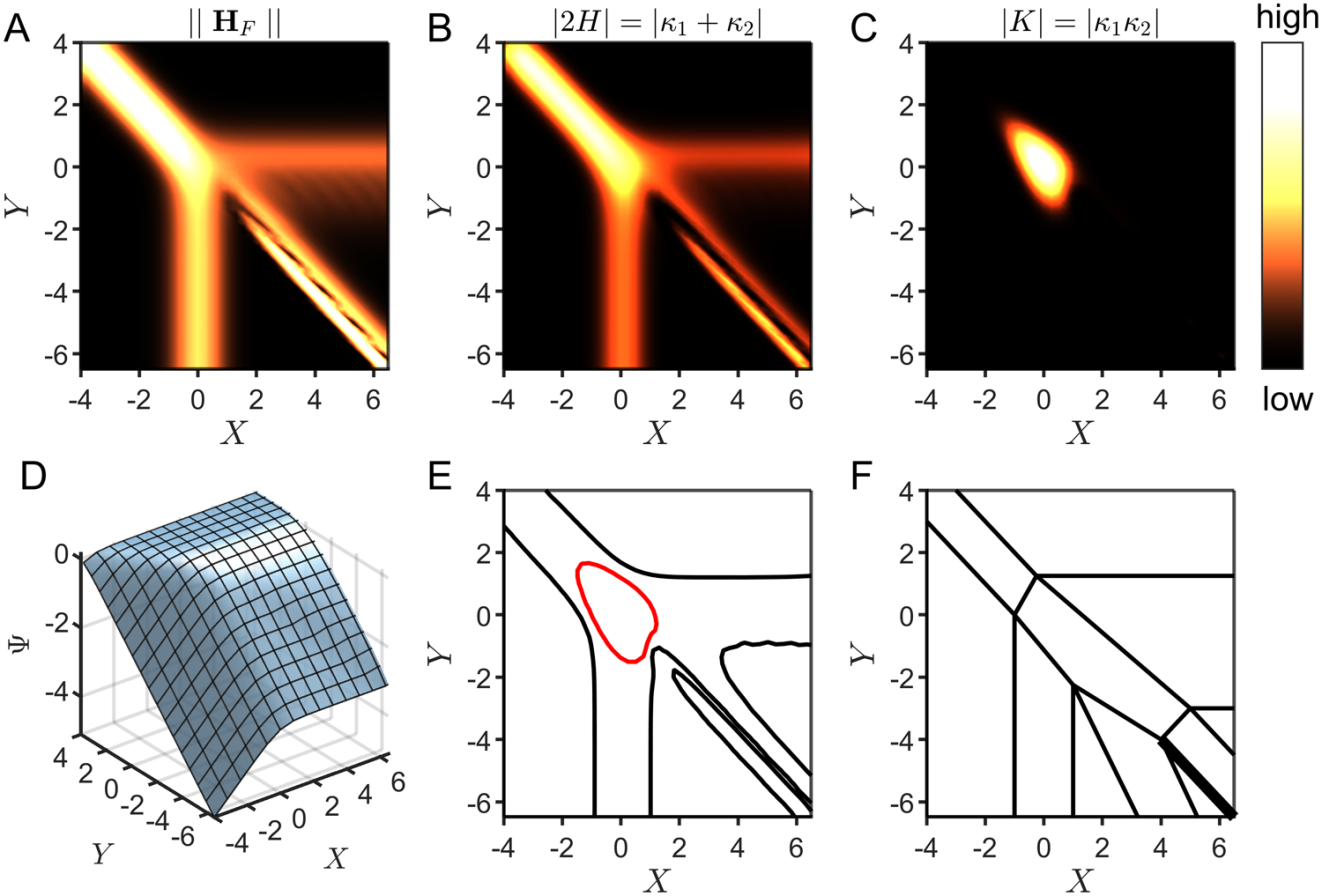
Case study of an **ECCE′** mechanism for homogeneous molecular CO_2_ reduction by an Fe(0) catalyst.^43^ Zone diagrams are visualized using both the quadratic approximation and curvature. The norm of the Hessian ∥**H**_*F*_∥ (A) and the absolute values of the mean curvature |2*H*| (B) and Gaussian curvature |*K*| (C) were used to construct heat maps over the two-dimensional parameter space (black regions correspond to low curvature and bright yellow regions correspond to high curvature). The values were obtained from the geometric surface (D) generated for this mechanism by using numerical methods to compute the steady-state current. A zone diagram (E) is built from a composite of the mean and Gaussian curvature, where the boundaries are defined as 20% of the maximum mean curvature (zero- and one-parameter zones, black lines) and 20% of the maximum Gaussian curvature (two-parameter zone, red line). (F) The reported zone diagram is displayed for comparison.^43^

We can define a two-dimensional parameter space with *X* = log κ and *Y* = log λ. The geometric surface, curvature plots, and resulting zone diagrams are displayed in Figure 8A–E. From the original report by Costentin and Savéant, an abrupt transition was noted in the zone diagram (reproduced in Figure 8F).^43^ This occurs in the bottom right-hand corner (indicated by the bold boundary in Figure 8F), where the binding of CO_2_ is essentially irreversible and the C–O bond-breaking step, consuming *B* and producing *C*, is rate-limiting. It was found that as CO_2_ binding becomes more and more irreversible (by increasing κ, which translates the system from left to right in a horizontal line on the zone diagram), the system rapidly changes from a situation where the intermediate *C* is at steady state to one where it is not at steady state.

This change in the mechanism is also captured by the curvature, where both ∥**H**_*F*_∥ and |2*H*| suddenly increase and then decrease again in a narrow region of the parameter space. We show this in the Supporting Information (Section 5, p. S13) that this sharp change is characteristic of triggering the onset of nonlinear behavior as the parameter κ is varied. Nonlinear kinetics are most often responsible for instability leading to abrupt transitions in dynamical systems.^17,47−49^

Furthermore, the zones spanning the transition between steady and nonsteady states cover a relatively small total area within the zone diagram. In fact, anywhere the absolute value of the curvature is relativity large, by definition, the corresponding zone will occupy only a very small area of the parameter space since highly curved regions transition rapidly between the limiting flat zones. We can conclude from this that narrow transition zones (one or two parameter zones) with high curvature are good indicators of the appearance of nonlinear behavior in an electrochemical system.

While the mechanistic switch from steady state to nonsteady state (linear to nonlinear kinetics) in this two-dimensional example was previously identified through careful application of analytical techniques,^43^ characterizing nonlinear transitions in more complex systems with larger dimensional parameter spaces (i.e., more than two governing parameters) may be challenging using traditional analytical or numerical methods (*vide infra*). A geometric approach using curvature is intrinsically able to identify such regions, and importantly, this scales to higher dimensions.

### Higher Dimensional Zone Diagrams

“All pleasures palled upon me; all sights tantalized and tempted me to outspoken treason, because I could not but compare what I saw in Two Dimensions with what it really was if seen in Three···”—excerpt from *Flatland: A Romance of Many Dimensions*.^50^

One advantage of this method is that it generalizes to higher dimensional parameter spaces. Many electrochemical systems will contain more than two governing dimensionless parameters, making it not possible to visualize the system with a two-dimensional zone diagram (remembering that the number of dimensionless groups determines the dimensionality of the resultant zone diagram). For example, the competition between ECE and disproportionation mechanisms in the context of coupled homogeneous reactions was previously investigated with a three-dimensional zone diagram.^51^ In the study of mediated enzymatic catalysis in a film, the model contained in total four governing parameters.^21^ A two-dimensional zone diagram could be constructed by holding two parameters constant while the remaining two formed the axes (dimensions) of the zone diagram. Because of the remaining two parameters, Figure 7E actually shows only a projection of the full multidimensional zone diagram onto the (*X*, *Y*) plane.^21^

**Figure 9:**
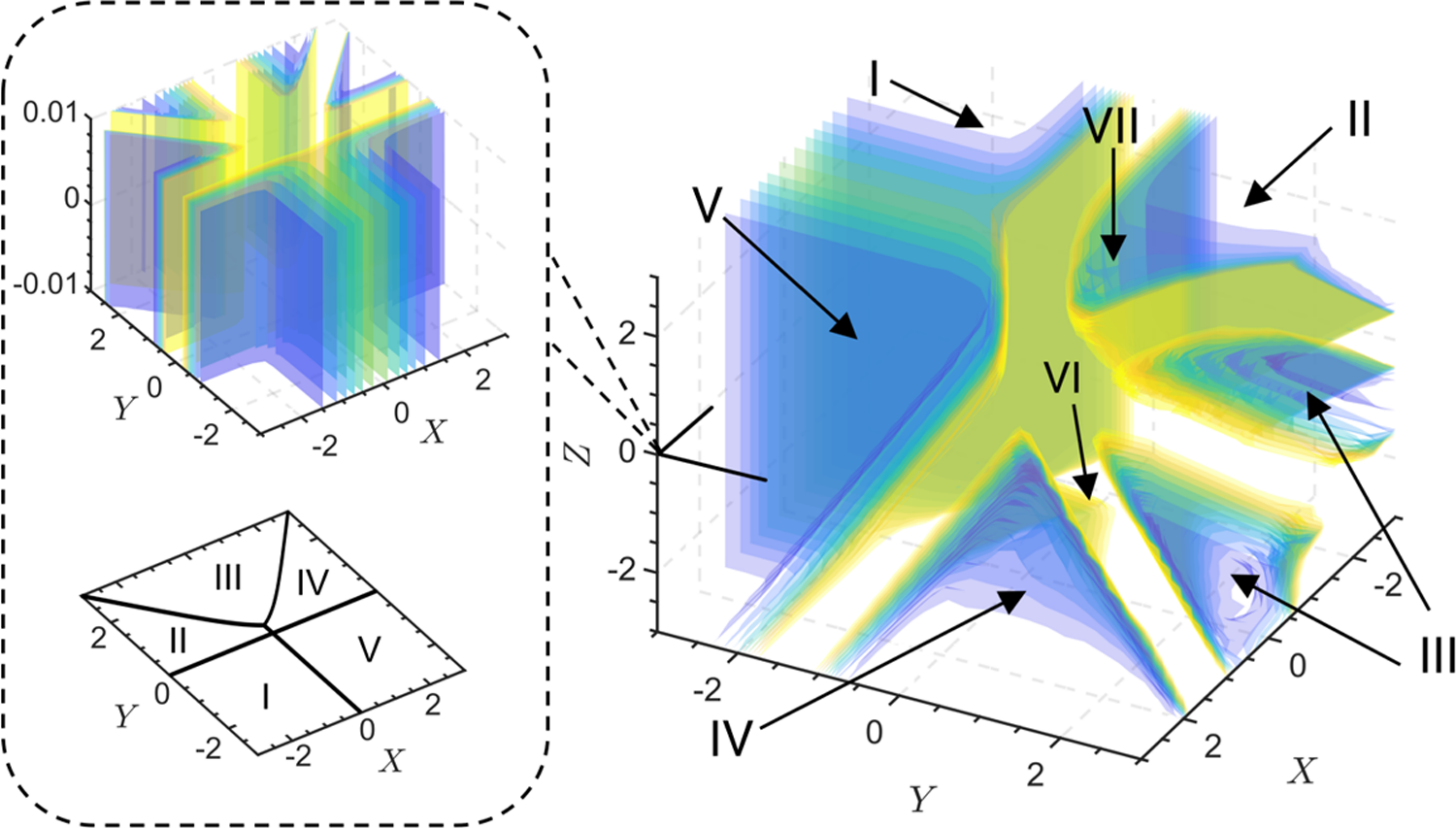
Three-dimensional zone diagram of enzymatic catalysis in a redox film.^21^ The axes are plotted with *X* = log γ, *Y* = log κ, and *Z* = log η (definitions of the dimensionless governing parameters are given in the Supporting Information, Section 4.2, p. S9). Colored planes are designated as level sets of ∥**H**_*F*_∥ (see Definition 1.4 and eq S5, Supporting Information). Blue regions signify limiting kinetic regimes (zero-parameter zones), and yellow regions correspond to transition regions (one-parameter and two-parameter zones). An inset shows the local structure around the *Z* = 0 plane, which matches the corresponding two-dimensional zone diagram. Zones are labeled according to the original nomenclature.^21^

Our geometric interpretation of zone diagrams allows us to examine the same system in three dimensions, now including a third parameter in the parameter space. The geometric surface obtained from the graph of the current function Ψ = *F*(*X*, *Y*, *Z*) is in fact now a three-dimensional manifold embedded in four-dimensional space (the exact definition of the third parameter *Z* is given in the Supporting Information, eqs S41 and S42). Zone boundaries are most easily visualized as level sets (the name for contours in higher dimensions) of ∥**H**_*F*_∥. Figure 9 displays the three-dimensional zone diagram for this system. An inset, enlarging the region around the *Z* = 0 plane, shows that the local structure corresponds to that of the reported zone diagram,^21^ where *Z* is set identically to zero.

Moving up and down the *Z* axis, new zones appear, while others shrink and fade away, emphasizing the multidimensional behavior of the system. This geometric analysis is completely general and can in principle be performed for systems with any number of  dimensionless groups, as dictated by Buckingham’s Π theorem, generating -dimensional zone diagrams.

### Discovering New Kinetic Behavior with Geometry

Solving the systems of partial or ordinary differential equations, mentioned in the introduction, cannot usually be done in close form due to the frequent appearance of nonlinear reaction terms. To make progress toward solving the system for the current response and constructing the corresponding zone diagram, one must then rely on simplifying assumptions to obtain usable solutions. At the outset, sometimes it is difficult to arrive at which assumptions to apply and what range of the dimensionless governing parameters is appropriate for a particular assumption, given the electrochemical system under investigation.

Conversely, the geometric framework presented here works in the opposite direction: it generates conditions that match with each limiting kinetic behavior and quantitatively predicts their corresponding area of validity in the parameter space. Implemented computationally, this process is automated and removes the uncertainty from the purely analytical method described above. However, a combined approach can provide significant benefits. One can first use geometry and curvature to numerically generate a desired zone diagram, and then, with information about the limiting kinetic regimes, apply the appropriate assumptions and solve the differential equations analytically (using techniques like perturbation and boundary layer theory)^52,53^ to yield very useful approximate asymptotic expressions for the current.

Embedded in this methodology is the potential to discover new kinetic behavior. Mapping the entire parameter space using curvature may reveal nuanced kinetic situations that occupy small regions of the parameter space. Such zones would perhaps be missed with analytical tools, which necessitates solving the differential equations manually. Brute-force numerical simulations, especially when implemented with dimensional variables (as is often the case), are also unlikely to uncover such zones since this essentially requires running an infinite number of “*in silico*” experiments.

This becomes exponentially more difficult when dealing with higher dimensional parameter spaces. We saw that zones with small areas are representative of abrupt transitions, typical of nonlinear kinetics, for example, the sharp switch from steady state to nonsteady state in the case study of homogeneous CO_2_ reduction (lower right corner of Figure 8E). These regions of instability in the parameter space translate to highly curved regions on the abstract geometric surface and often signify interesting or important physical behavior (for example, pattern formation, phase transitions, bifurcations, autocatalytic reactions, etc.). Ascertaining the location of these zones within a multidimensional parameter space is significantly more straightforward by analyzing curvature. Combining automated discovery of kinetic regimes, enabled by the geometric framework presented here, with AI tools, such as physics-informed machine learning^54−56^ or neural networks,^57,58^ presents an interesting frontier for modeling electrochemical systems.

## Conclusions

Once the current output of an electrochemical system of interest is interpreted as a geometric surface, analyzing the geometry of this abstract surface will locate zone boundaries within the parameter space and enable the automated construction of zone diagrams. This procedure can be carried out computationally and is straightforward to implement with numerical simulations. Furthermore, the method could be applied to a number of important electrochemical systems from hetero- and homogeneous electrocatalysis.

However, the connection between zone diagrams and geometry goes well beyond the surface level (pun intended). For example, we discovered new definitions for the classification of kinetic zones in electrochemistry based on local topological equivalence to basic geometric objects such as cylinders and spheres. This provides a deeper understanding of kinetic behavior. We found that the method is able to discover new kinetic regimes, identify nonlinear behavior and instabilities in the parameter space, and extend the utility of zone diagrams to large dimensional systems. Although different in execution, this strategy to simplify a hard problem by using geometric reasoning is reminiscent of phase plane analysis from nonlinear dynamics.^59^

Electrochemistry is entering an exciting new era of discovery,^60^ corresponding to its direct importance in a sustainable energy transition. As a result, employing both numerical and analytical modeling for an ever-growing number of unique systems is an important endeavor. We hope the geometric interpretation of zone diagrams outlined here will enable systematic optimization and rational design of complex electrochemical systems encoded in geometry.

## Supporting Information Available

Additional model information for case studies: governing equations and numerical methods; definitions, procedures, and MATLAB code used for calculating curvature; and mathematical definitions of zone boundaries (PDF)
