# Supplementary material for: A Geometric Interpretation of Kinetic Zone Diagrams in Electrochemistry
Source: J Am Chem Soc. 2024 Dec 4;146(50):34771–85. doi: 10.1021/jacs.4c13271 (PMC11664506; doi:10.1021/jacs.4c13271)
Supplement: Supplementary file 1 — ja4c13271_si_001.pdf [file ja4c13271_si_001.pdf]

Supporting Information

A Geometric Interpretation of Kinetic Zone Diagrams in Electrochemistry

Nicolas Plumeré & Ben A. Johnson*

Technical University of Munich (TUM), Campus Straubing for Biotechnology and Sustainability, Uferstraße 53, 94315 Straubing, Germany

*Corresponding Author: Ben A. Johnson
Email: ben.johnson@tum.de

Contents

1 Zone boundary definitions **S3**
1.1 Equated boundaries S3
1.2 Percent error boundaries S3
1.3 Boundaries based on curvature S3

2 Introduction to asymptotics **S4**
2.1 Application to zone diagrams S4
2.2 Zero, one, and two parameter zones S4

3 Calculation of curvature **S5**

4 Case Studies: continuum models of electrochemical systems **S7**
4.1 Electrocatalytic films S7
4.2 Mediated enzymatic catalysis in a redox film S9
4.3 CO_2 reduction by a homogeneous molecular Fe(0) catalyst S11

5 Curvature analysis of nonlinear behavior **S13**

6 Methods **S15**
6.1 Numerical methods S15

Author Contributions **S18**

References **S18**

Figures

S1 Example of a parameterized surface S5
S2 Schematic of electrocatalytic films S8
S3 Schematic of mediated enzymatic catalysis in a redox-polymer film S9
S4 Schematic of catalytic **ECCE′** mechanism S11
S5 Curvature analysis of nonlinear behavior S14
S6 Spatial grids for numerical solutions S17

1 Zone boundary definitions

1.1 Equated boundaries

Definition 1.1. Consider two distinct asymptotic approximations (see Section 2 for more details on asymptotic approximations) for the current $\psi_i^{\text{asym}}(x, y)$ and $\psi_j^{\text{asym}}(x, y)$ defined on the open set $D = \{(x, y) \in \mathbb{R}^2 \mid x > 0,\, y > 0\}$ with two dimensionless governing parameters x and y. Using this method, the boundary between these zones is the set of governing parameters given by

$$Z_{ij} = \{(x, y) \in D \mid \psi_i^{\text{asym}}(x, y) = \psi_j^{\text{asym}}(x, y)\}. \tag{S1}$$

1.2 Percent error boundaries

Definition 1.2. Let ψ^{num} be a global solution for the current response calculated numerically. As before, it will depend on the governing dimensionless parameters x and y. Additionally, ε is a constant representing the desired value of percent error. If ψ_k^{asym} is an asymptotic solution (this could be a zero *or* one parameter zone) defined on the open set $D = \{(x, y) \in \mathbb{R}^2 \mid x > 0,\, y > 0\}$, then the boundary for the zone corresponding to ψ_k^{asym} is given by

$$Z_k = \left\{(x, y) \in D \;\middle|\; \left|\frac{\psi^{\text{num}}(x, y) - \psi_k^{\text{asym}}(x, y)}{\psi^{\text{num}}(x, y)}\right| = \varepsilon\right\}. \tag{S2}$$

1.3 Boundaries based on curvature

Here, we introduce new definitions for zone boundaries based on level sets (or contours) of the mean curvature $2H$, the Gaussian curvature K, and the norm of the Hessian matrix $\|\mathbf{H}_F\|$. The variables on a logarithmic scale from the main text ($X = \log x$, $Y = \log y$, ...) are used. First, we define zone boundaries in two dimensions by the mean and Gaussian curvatures (Definition 1.3).

Definition 1.3. Let $\mathbf{X} \in \mathbb{R}^2$ be a vector containing the dimensionless governing parameters, $\mathbf{X} = (X, Y)$. If $F : \mathbb{R}^2 \to \mathbb{R}$ is the current function, then its graph is a two-dimensional manifold in three-dimensional space defined by the map $S : \mathbf{X} \mapsto (\mathbf{X}, F(\mathbf{X}))$. Let $p = (\mathbf{X}, F(\mathbf{X}))$ be a point on S. We introduce the functions $\mathcal{K}(\mathbf{X}) = K(p)$ and $\mathcal{H}(\mathbf{X}) = H(p)$ that relate the curvature at a point p on S to the corresponding point $\mathbf{X}$ in the parameter space, where $K(p)$ is the Gaussian curvature and $H(p)$ is the mean curvature at point p. If $\mathcal{C} \in \mathbb{R}$ is a constant, the zone boundaries are defined as

$$Z_K = \{\mathbf{X} \in \mathbb{R}^2 \mid \mathcal{K}(\mathbf{X}) = \mathcal{C}\}, \tag{S3}$$

$$Z_H = \{\mathbf{X} \in \mathbb{R}^2 \mid \mathcal{H}(\mathbf{X}) = \mathcal{C}\}. \tag{S4}$$

Next, to show the generality of this approach, we will also include any number of dimensionless governing parameters in the definition, corresponding to a general $\mathcal{N}$-dimensional zone diagram. Zone boundaries in higher dimensions can be defined straightforwardly from the quadratic approximation as a level set of the norm of the Hessian matrix (Definition 1.4).

Definition 1.4. Let $\mathbf{X} \in \mathbb{R}^{\mathcal{N}}$ be a vector representing the dimensionless governing parameters, with $\mathcal{N}$ denoting the number of such parameters, and let $F : \mathbb{R}^{\mathcal{N}} \to \mathbb{R}$ be the current function. The graph of F defines an $\mathcal{N}$-dimensional manifold in $\mathbb{R}^{\mathcal{N}+1}$, given by the map $S : \mathbf{X} \mapsto (\mathbf{X}, F(\mathbf{X}))$. If $\mathcal{C} \in \mathbb{R}$ is a constant, the zone boundaries are defined as

$$Z_Q = \left\{\mathbf{X} \in \mathbb{R}^{\mathcal{N}} \;\middle|\; \|\mathbf{H}_F(\mathbf{X})\| = \mathcal{C}\right\}, \tag{S5}$$

where $\mathbf{H}_F(\mathbf{X})$ denotes the Hessian matrix of F evaluated at $\mathbf{X}$.

2 Introduction to asymptotics

Two functions, f and g, are *asymptotic* [1] when

$$\lim_{x \to x_0} \frac{f(x)}{g(x)} = 1. \tag{S6}$$

This is written using the "$\sim$" symbol as

$$f(x) \sim g(x) \qquad \text{"}f \text{ is asymptotic to } g \text{ as } x \to x_0.\text{"} \tag{S7}$$

The asymptotic relationship is always defined in the limit as a variable or parameter approaches a particular value ($x \to x_0$), typically $x \to 0$ or $x \to \infty$.

2.1 Application to zone diagrams

Here we provide a proof to show that in the limit of very large or small values for the dimensionless governing parameters the current function always reduces to a power law monomial:

$$\psi(x, y) \sim \alpha x^a y^b. \tag{S8}$$

To validate this statement, let's look at the situation where both dimensionless governing parameters are very small, $(x, y) \to (0, 0)$, as an example. Although, the following reasoning also applies when either are large, since the inverse of a large number is itself small. Leveraging the smallness of x and y allows us to use an *asymptotic expansion* [2] to approximate the current function. Luckily, only the first few terms of these series are typically required for a very accurate approximation when one or more parameters in the problem tend towards a small number. Perturbation theory utilizes this same strategy, [1, 2] and a common example of an asymptotic expansion is a simple power series. Expanding the current function, first in terms of powers of x, yields

$$\psi(x, y) \sim x^{a_0}\psi_0(y) + x^{a_1}\psi_1(y) + x^{a_2}\psi_2(y) + \ldots, \tag{S9}$$

as $x \to 0$, where the a_i's are a series of constants, and $\psi_i(y)$ are functions of y. We retain only the leading order term, ψ_0, and then apply a similar expansion, now in terms of powers of y. The result is

$$\psi_0(y) \sim y^{b_0}\psi_{0,0} + y^{b_1}\psi_{0,1} + y^{b_2}\psi_{0,2} + \ldots, \tag{S10}$$

as $x \to 0$ and $y \to 0$, where b_j are constant powers, and the coefficients $\psi_{i,j}$ are also constants with respect to x and y. Taking again the lowest order term and combining these two results, we obtain

$$\psi(x, y) \sim x^{a_0} y^{b_0} \psi_{0,0} + \mathcal{O}(a_1, b_1), \tag{S11}$$

where the remaining coefficient, $\psi_{0,0}$, is a constant (it does not depend on x or y), and $\mathcal{O}(a_1, b_1)$ is the error term associated with making these approximations. This result, obtained by using reasoning from asymptotic approximations and perturbation theory, says that in the extreme limits of x and y, the current function can always be approximated by an expression that is in the form of a power law monomial.

2.2 Zero, one, and two parameter zones

In fact, the analysis above also provides a definition for zero, one, and two parameter zones, summarized in the table below. A central two parameter zone is characterized by the exact form of the current function $\psi(x, y)$. Typically, it is sufficient to assume that the lowest order term in an asymptotic series corresponds to the unperturbed problem (where the small parameter is set identically to zero) [1, 2], and we take $a_0 = b_0 = 0$. The first asymptotic expansion is representative of the transition to a one parameter zone as $x \to 0$. The resulting leading-order

asymptotic expression for the current $\psi_0(y)$, indeed, only depends on one parameter, y. The composition with a second asymptotic expansion, now as $x \to 0$, $y \to 0$, yields a zero parameter zone, where the asymptotic expression for the current $\psi_{0,0}$ does not depend on any parameters and is a constant with respect to both x and y.

	zero-parameter zone	one-parameter zone	two-parameter zone
expression	$\psi_{0,0}$	$\psi_0(y)$	$\psi(x, y)$
parameters	constant	y	x and y
number of parameters	0	1	2

3 Calculation of curvature

The curvature of a surface can be defined using differential geometry [3]. Let $D \in \mathbb{R}^2$ be the domain of a smooth function $f : D \to \mathbb{R}$. Then the graph of f is a parameterized surface given by $\sigma(x, y) = (x, y, f(x, y))$ (Figure S1). For compatibility with computations, we use the local

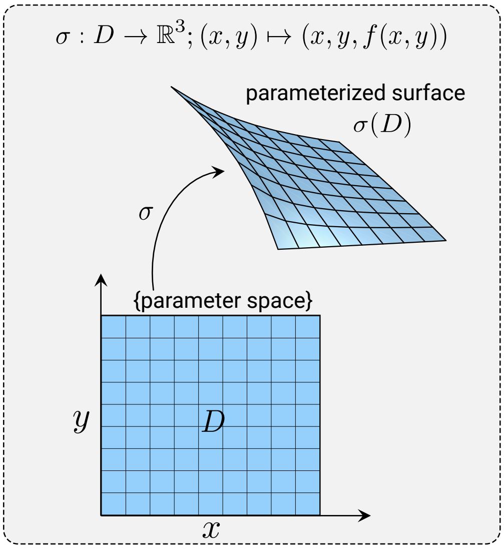

Figure S1. The transformation σ takes points in D into three dimensions producing an image $\sigma(D)$, which is an equivalent representation of the surface obtained by graphing the current function. Points on the surface are located at $(x, y, f(x, y))$, where $\psi = f(x, y)$.

coordinate expressions for the first and second fundamental forms, from which Gaussian and mean curvature can be calculated. The coefficients of the first fundamental form are

$$L = 1 + f_x^2, \qquad M = f_x f_y, \qquad N = 1 + f_y^2. \tag{S12}$$

The notation $f_x = \partial f / \partial x$ is used here for partial derivatives. The coefficients of the second fundamental form are

$$l = \frac{f_{xx}}{\sqrt{1 + f_x^2 + f_y^2}}, \qquad m = \frac{f_{xy}}{\sqrt{1 + f_x^2 + f_y^2}}, \qquad n = \frac{f_{yy}}{\sqrt{1 + f_x^2 + f_y^2}}. \tag{S13}$$

The Gaussian curvature at any point is

$$K = \frac{ln - m^2}{LN - M^2} = \frac{f_{xx} f_{yy} - f_{xy}^2}{\left(1 + f_x^2 + f_y^2\right)^2}. \tag{S14}$$

The mean curvature is calculated from

$$2H = \frac{lN - 2mM + mL}{LN - M^2} = \frac{f_{xx}\left(1+f_y^2\right) - 2f_{xy}f_xf_y + f_{yy}\left(1+f_x^2\right)}{\left(1+f_x^2+f_y^2\right)^{3/2}}. \tag{S15}$$

We note that the mean curvature is extrinsic and therefore still a local measurement of curvature. However, because the condition $H \neq 0$ discriminates between flat and curved regions on our abstract electrochemical surface to uniquely identify one parameter zones (transition zones that only depend on one governing parameter), it is a useful metric for our purposes.

To visualize zone diagrams using the quadratic approximation, the Frobenius or Euclidian matrix norm was used in combination with the Hessian matrix, defined as

$$\mathbf{H}_f = \begin{bmatrix} f_{xx} & f_{xy} \\ f_{yx} & f_{yy} \end{bmatrix}. \tag{S16}$$

In any number of dimensions, if $\mathbf{H}_f$ is a $\mathcal{N} \times \mathcal{N}$ matrix with elements h_{ij}, its norm is defined by

$$\|\mathbf{H}_f\| = \sqrt{\sum_{i=1}^{\mathcal{N}} \sum_{j=1}^{\mathcal{N}} |h_{ij}|^2}. \tag{S17}$$

Calculations for the norm of the Hessian, mean curvature, and Gaussian curvature were implemented computationally using the following MATLAB code:

```
function [normH] = findHessian(x,y,fxn)

    % find grid spacing
    dx = abs(diff(x(1:2)));
    dy = abs(diff(y(1:2)));

    % compute first and second partial derivatives
    [fx, fy] = gradient(fxn,dx,dy);
    [fxx, fxy] = gradient(fx,dx,dy);
    [fyx, fyy] = gradient(fy,dx,dy);

    for j = 1:length(fxx)
        for i = 1:length(fxx)
            H = [fxx(i,j) fyx(i,j);
                fxy(i,j) fyy(i,j)];

            normH(i,j) = norm(H,"fro"); % Frobenius norm
        end
    end
end
```

```
function [normH] = findHessian3D(x,y,z,fxn)

    % find grid spacing
    dx = abs(diff(x(1:2)));
    dy = abs(diff(y(1:2)));
    dz = abs(diff(z(1:2)));

    % compute first and second partial derivatives
    [fx, fy, fz] = gradient(fxn,dx,dy,dz);
    [fxx, fxy, fxz] = gradient(fx,dx,dy,dz);
    [fyx, fyy, fyz] = gradient(fy,dx,dy,dz);
    [fzx, fzy, fzz] = gradient(fz,dx,dy,dz);

    for j = 1:length(fxx)
        for i = 1:length(fxx)
            for k = 1:length(fxx)
```

```
                H = [fxx(i,j,k) fyx(i,j,k) fzx(i,j,k);
                     fxy(i,j,k) fyy(i,j,k) fzy(i,j,k)
                     fxz(i,j,k) fyx(i,j,k) fzz(i,j,k)];
                normH(i,j,k) = norm(H,"fro"); % Frobenius norm
            end
        end
    end
end
```

```
function [gaussianCurvature, meanCurvature] = findCurvature(x,y,fxn)

    % find grid spacing
    dx = abs(diff(x(1:2)));
    dy = abs(diff(y(1:2)));

    % compute first and second partial derivatives
    [fx, fy] = gradient(fxn,dx,dy);
    [fxx, fxy] = gradient(fx,dx,dy);
    [fyx, fyy] = gradient(fy,dx,dy);

    % compute coefficients of the First Fundamental form
    L = 1 + fx.^2;
    M = fx.*fy;
    N = 1 + fy.^2;

    % compute coefficients of the Second Fundamental form
    l = fxx./(sqrt(1 + fx.^2 + fy.^2));
    m = fxy./(sqrt(1 + fx.^2 + fy.^2));
    n = fyy./(sqrt(1 + fx.^2 + fy.^2));

    gaussianCurvature = (l.*n - m.^2)./(L.*N - M.^2);
    meanCurvature = (l.*N - 2.*m.*M + m.*L)./(L.*N - M.^2);

end
```

4 Case Studies: continuum models of electrochemical systems

4.1 Electrocatalytic films

$$\mathrm{P} + e^- \leftrightharpoons \mathrm{Q}$$

$$\mathrm{Q} + \mathrm{S} \xrightarrow{k_1} \mathrm{P} + \text{product}$$

Here we provide a brief summary of the continuum model for an electrocatalytic film with a simple one-step, one-electron reaction as previously reported [4]. Consider an electrocatalytic film of thickness ℓ (Figure S2). An electrochemical reaction at the interface ($x = 0$) generates an active catalytic species Q. Charge transport through the film takes place as an equivalent diffusion process with a diffusivity given by D_e. The substrate S diffuses through the film, with a diffusion coefficient D_S, and the reaction proceeds with a second-order rate constant k_1. Under pure kinetic conditions (steady state with respect to time), the governing equations ($0 < x < \ell$) are

$$D_\mathrm{e}\frac{d^2Q}{dx^2} - k_1 QS = 0, \tag{S18}$$

$$D_\mathrm{S}\frac{d^2S}{dx^2} - k_1 QS = 0. \tag{S19}$$

Negligible substrate depletion in the electrolyte solution is assumed such that the concentration of substrate at the film-solution interface is equal to its bulk value. This can be achieved by

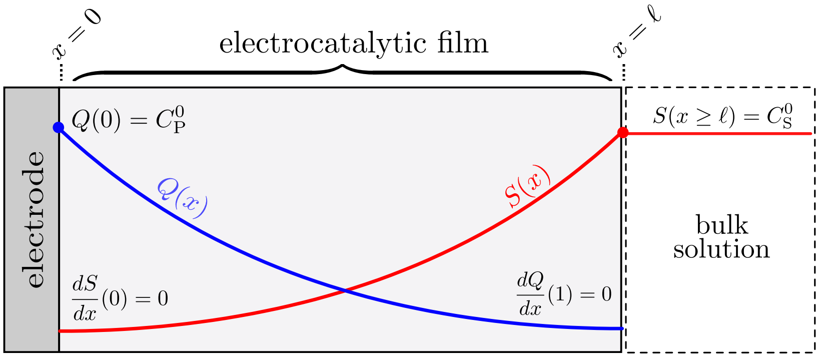

Figure S2. Schematic of a one-dimensional electrocatalytic film interfaced with an electrolyte solution [4].

employing forced convection in the electrolyte solution outside the film, short timescales, and/or a relatively large substrate concentration. Additionally, the substrate is not directly reduced at the electrode-film interface in the potential window of interest, giving a corresponding no-flux condition. We also take the applied electrode potential E to be more negative of that of the standard potential of the catalyst E^0, such that $\exp\big(F/RT(E - E^0)\big) \ll 1$, and the surface concentration of the active (reduced) catalyst Q at the electrode is equal to the total concentration of catalytic active sites C_P^0. This allows us to observe the plateau current. For simplicity, the transport of substrate across the film-solution interface is assumed to be at a fast equilibrium, and any partition coefficients are equal to unity. This leads to the following boundary conditions for the electrode surface ($x = 0$) and the film-solution interface ($x = \ell$):

$$Q(0) = C_\mathrm{P}^0, \quad \frac{dQ}{dx}(\ell) = 0, \tag{S20}$$

$$S(\ell) = C_\mathrm{S}^0, \quad \frac{dS}{dx}(0) = 0. \tag{S21}$$

Steady-state plateau current is defined as either the flux of the reduced catalyst at the electrode-film interface ($x = 0$) or the flux of the substrate at the film-solution interface ($x = \ell$):

$$\frac{i}{SF} = -D_\mathrm{e}\frac{dQ}{dx}(0) = D_\mathrm{S}\frac{dS}{dx}(\ell), \tag{S22}$$

where S is the electrode surface area, and F is Faraday's constant. We now introduce the following dimensionless variables

$$p = \frac{P}{C_\mathrm{P}^0}, \qquad q = \frac{Q}{C_\mathrm{P}^0}, \qquad s = \frac{S}{C_\mathrm{S}^0}, \qquad y = \frac{x}{\ell}, \qquad \psi = \frac{i}{FSk_1C_\mathrm{P}^0C_\mathrm{S}^0\ell}.$$

This results in a dimensionless system of ordinary differential equations:

$$\frac{d^2q}{dy^2} - \left(\frac{1}{\lambda_\mathrm{e}}\right)^2 qs = 0, \tag{S23}$$

$$\frac{d^2s}{dy^2} - \left(\frac{1}{\lambda_\mathrm{s}}\right)^2 qs = 0. \tag{S24}$$

The boundary conditions in dimensionless form become

$$q(0) = 1, \quad \frac{dq}{dy}(1) = 0, \tag{S25}$$

$$s(1) = 1, \quad \frac{ds}{dy}(0) = 0, \tag{S26}$$

and the dimensionless current is

$$\psi = -\lambda_e^2 \frac{dq}{dy}(0) = \lambda_s^2 \frac{ds}{dy}(1). \tag{S27}$$

Two dimensionless groups govern the system; we define these as

$$\lambda_e = \frac{1}{\ell}\sqrt{\frac{D_e}{k_1 C_S^0}}, \qquad \lambda_s = \frac{1}{\ell}\sqrt{\frac{D_s}{k_1 C_P^0}}. \tag{S28}$$

The form in which these dimensionless parameters were used in the original zone diagram [5] is equivalent to the inverse of the *Thiele modulus*, a notable dimensionless group from chemical reaction engineering [6]. In the original formulation, each process (charge transport, substrate diffusion, and the catalytic reaction) was associated with a characteristic current density (i_e, i_s, and i_k, respectively) [4, 7]. Ratios of these current densities are dimensionless groups and can be used as an alternative definition for the two governing parameters. With the dimensionless groups in hand, the parameter space can be defined. On a logarithmic scale we obtain

$$X = \log \lambda_e, \qquad Y = \log \lambda_s. \tag{S29}$$

Finally, the current function becomes

$$\Psi(X, Y) = \log\left(\psi(\lambda_e, \lambda_s)\right). \tag{S30}$$

4.2 Mediated enzymatic catalysis in a redox film

$$\mathrm{P} + e^- \leftrightharpoons \mathrm{Q}$$
$$\mathrm{Q} + \mathrm{E_1} \xrightarrow{k_e} \mathrm{E_2} + \mathrm{P}$$
$$\mathrm{E_2} + \mathrm{S} \xrightarrow{k_{\mathrm{cat}},\, K_{\mathrm{M}}} \mathrm{E_1} + \text{product}$$

The model for mediated enzymatic catalysis in a redox film (Figure S3), as previously reported [8], is briefly summarized. Enzymes within a film of thickness ℓ are globally immobile with a total concentration C_E^0, where E_1 is the oxidized state and E_2 is the reduced state. Charge transfer

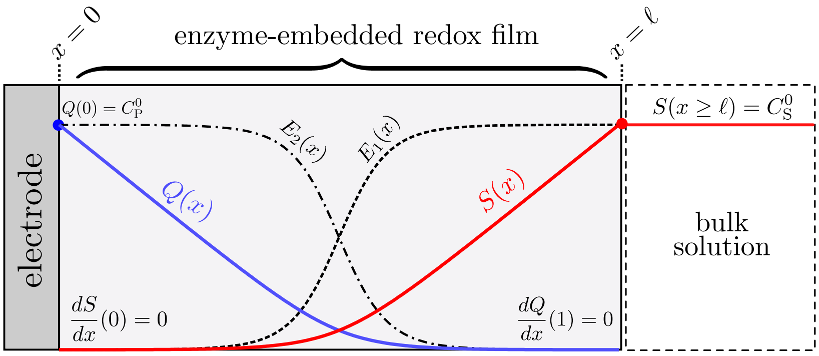

Figure S3. Schematic of a redox-polymer film for mediated enzymatic catalysis, where S is the substrate, Q is the reduce mediator, E_1 is the oxidized state of the enzyme, and E_2 is the reduced state of the enzyme [8].

takes place through a formally diffusion process (electron hopping between mediators P/Q) with an equivalent diffusion coefficient D_e. The total mediator concentration is C_P^0. Outersphere electron transfer between the enzyme and mediator occurs with a simple second order rate consant k_e. Further, the enzymatic reaction is described by typical Michaelis-Menten kinetics

(k_{cat}, K_{M}), and the electrode potential is set to provide enough overpotential such that the reduced mediator concentration at the electrode-film interface $Q(0)$ is equal to C_{P}^{0}. At timescales longer that then global timescale for the reactions, the processes within the film are at a pseudo-state state, and a steady-state plateau current can be observed. It is further assumed that the mediator concentration is in large excess to that of the enzyme, resulting in a pseudo-steady approximation for the immobile enzyme species. This, in combination with the fact that both the enzyme concentration and mediator concentration are conserved scalar quantities ($P(x) + Q(x) = C_{\mathrm{P}}^{0}$ and $E_1(x) + E_2(x) = C_{\mathrm{E}}^{0}$), results in a system of two equations in terms of Q and S ($0 < x < \ell$):

$$D_{\mathrm{e}}\frac{d^2Q}{dx^2} - \frac{k_{\mathrm{e}}k_{\mathrm{cat}}C_{\mathrm{E}}^{0}QS}{k_{\mathrm{e}}Q\left(K_{\mathrm{M}} + S\right) + S} = 0, \tag{S31}$$

$$D_{\mathrm{S}}\frac{d^2S}{dx^2} - \frac{k_{\mathrm{e}}k_{\mathrm{cat}}C_{\mathrm{E}}^{0}QS}{k_{\mathrm{e}}Q\left(K_{\mathrm{M}} + S\right) + S} = 0. \tag{S32}$$

The concentrations of E_1 and E_2 are still, however, functions of the spatial variable x through their dependence on Q and S. Forced-convection maintains the substrate concentration at the film-solution interface at its bulk value C_{S}^{0}. For simplicity we consider partitioning of the substrate into the film a fast equilibrium such that any partition coefficients are unity. The relevant boundary conditions are

$$Q(0) = C_{\mathrm{P}}^{0}, \quad \frac{dQ}{dx}(\ell) = 0, \tag{S33}$$

$$S(\ell) = C_{\mathrm{S}}^{0}, \quad \frac{dS}{dx}(0) = 0. \tag{S34}$$

Steady-state plateau current is defined as either the flux of the mediator at the electrode-film interface ($x = 0$) or the flux of the substrate at the film-solution interface ($x = \ell$):

$$\frac{i}{SF} = -D_{\mathrm{e}}\frac{dQ}{dx}(0) = D_{\mathrm{S}}\frac{dS}{dx}(\ell). \tag{S35}$$

We now introduce the following dimensionless variables

$$p = \frac{P}{C_{\mathrm{P}}^{0}}, \qquad q = \frac{Q}{C_{\mathrm{P}}^{0}}, \qquad s = \frac{S}{C_{\mathrm{S}}^{0}}, \qquad y = \frac{x}{\ell}, \qquad \psi = \frac{i}{FSC_{\mathrm{P}}^{0}\frac{D_{\mathrm{e}}}{\ell}}.$$

This results in a dimensionless system of ordinary differential equations:

$$\frac{d^2q}{dy^2} - \frac{\kappa^2 qs}{\gamma q\left(1 + \mu s\right) + s} = 0, \tag{S36}$$

$$\frac{d^2s}{dy^2} - \frac{\kappa^2\gamma\eta^{-1}qs}{\gamma q\left(1 + \mu s\right) + s} = 0. \tag{S37}$$

The boundary conditions in dimensionless form become

$$q(0) = 1, \quad \frac{dq}{dy}(1) = 0, \tag{S38}$$

$$s(1) = 1, \quad \frac{ds}{dy}(0) = 0, \tag{S39}$$

and the dimensionless current is

$$\psi = -\frac{dq}{dy}(0) = \eta\gamma^{-1}\frac{ds}{dy}(1). \tag{S40}$$

Four dimensionless groups govern the system:

$$\kappa = \ell\sqrt{\frac{k_e C_E^0}{D_e}}, \quad \gamma = \frac{k_e C_P^0 K_M}{k_{cat} C_S^0}, \quad \mu = \frac{C_S^0}{K_M}, \quad \eta = \frac{D_S k_e K_M}{D_e k_{cat}}. \tag{S41}$$

We are interested in visualizing two-dimensional and three-dimensional projections of the total four-dimensional parameter space as zone diagrams. Therefore we set $\mu = 0.01$ as a global constant, and define

$$X = \log\gamma, \qquad Y = \log\gamma, \qquad Z = \log\eta. \tag{S42}$$

The current function is

$$\Psi(X, Y, Z) = \log\left(\psi(\gamma, \kappa, \eta)\right). \tag{S43}$$

For the two dimensional zone diagram in Figure 7e in the main text, the third parameter Z is set to a constant $Z = 0$. This means that points are represented as $(X, Y, 0)$, allowing us to look at a projection of three-dimensional space onto the (X, Y) plane.

4.3 CO_2 reduction by a homogeneous molecular Fe(0) catalyst

$$\mathrm{P} + e^- \leftrightharpoons \mathrm{Q}$$
$$\mathrm{Q} + \mathrm{S} \underset{k_{-1}}{\overset{k_1}{\rightleftarrows}} \mathrm{B}$$
$$\mathrm{B} \xrightarrow{k_2} \mathrm{C}$$
$$\mathrm{Q} + \mathrm{C} \xrightarrow{k_3} 2\,\mathrm{P} + \text{product}$$

Here we consider the formal **ECCE′** mechanism, [9] corresponding to that of homogeneous molecular CO_2 reduction by a freely diffusing catalyst (Figure S4). In the mechanism above,

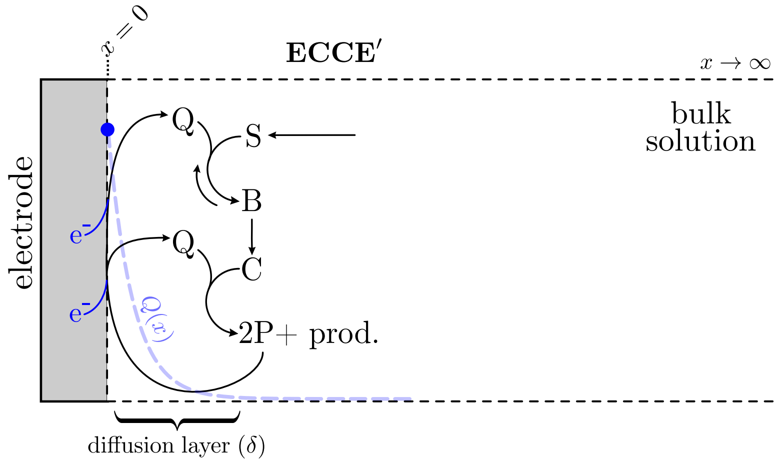

Figure S4. Schematic of a homogeneous catalytic **ECCE′** mechanism, representative of CO_2 reduction by molecular Fe(0) catalysts [9].

CO_2 binding (the reaction between Q and S) is described by a pseudo first order rate constant k_1, where the substrate concentration is constant throughout the experiment. All the intermediates diffuse freely in solution with a diffusion coefficient D. Ensuring pure kinetic conditions (pseudo-steady state) requires the timescale of the experiment to be longer than the global timescale of the coupled chemical reactions. In cyclic voltammetry, this is accomplished when $\lambda = F/RT(k/\nu)$ is large, where k is the relevant catalytic rate constant and ν is the scan rate. Additionally, we assume negligible depletion of the substrate S in the diffusion layer, which is valid for relatively

large bulk substrate concentrations (compared to that of the catalyst) and/or fast scan rates. This ensures the system is globally pseudo steady and we are able to observe the stead-state plateau current. Thus, we have the following system of ordinary differential equations

$$D\frac{d^2P}{dx^2} + 2k_3QC = 0, \tag{S44}$$

$$D\frac{d^2Q}{dx^2} - k_1Q + k_{-1}B - k_3QC = 0, \tag{S45}$$

$$D\frac{d^2B}{dx^2} + k_1Q - (k_{-1} + k_2)B = 0, \tag{S46}$$

$$D\frac{d^2C}{dx^2} + k_2B - k_3QC = 0. \tag{S47}$$

The total concentration of catalyst present is given by C^0_P. Initially, only P is present and the concentration of all other catalytic intermediates is zero. This system has a semi-infinite right-hand boundary condition given by

$$\lim_{x\to\infty} P = C^0_\mathrm{P} \tag{S48}$$

For all other intermediates C_j (j = Q, B, C), the initial conditions are maintained in the bulk solution, resulting in

$$\lim_{x\to\infty} C_j = 0. \tag{S49}$$

Mass conservation at the electrode surface reveals both the concentration of all catalytic species and their respective fluxes are conserved scalar quantities:

$$P(0) + Q(0) + B(0) + C(0) = C^0_\mathrm{P}, \tag{S50}$$

$$\frac{dP}{dx}(0) + \frac{dQ}{dx}(0) + \frac{dB}{dx}(0) + \frac{dC}{dx}(0) = 0. \tag{S51}$$

The steady-state plateau current is defined as

$$\frac{i}{SF} = -D\frac{dQ}{dx}(0). \tag{S52}$$

Additionally, the applied electrode potential E is set to be more negative of that of the standard potential of the catalyst E^0, such that $\exp\big(F/RT(E - E^0)\big) \ll 1$, leading to a catalytic plateau. The system of equation and boundary conditions are suitably non-dimensionalized according to

$$j = \frac{C_j}{C^0_\mathrm{P}}, \qquad y = \frac{x}{\sqrt{\frac{D}{k_1}}}, \qquad \psi = \frac{i}{SFC^0_\mathrm{P}\sqrt{Dk_1}}.$$

The spatial variable x is normalized to the diffusion layer thickness $\delta = \sqrt{D/k_1}$ of the reduced catalyst Q. This results in the dimensionless system of equations

$$\frac{d^2p}{dy^2} + 2\rho qc = 0, \tag{S53}$$

$$\frac{d^2q}{dy^2} - q + \kappa^{-1}b - \rho qc = 0, \tag{S54}$$

$$\frac{d^2b}{dy^2} + q - (\kappa^{-1} + \lambda)b = 0, \tag{S55}$$

$$\frac{d^2c}{dy^2} + \lambda b - \rho qc = 0, \tag{S56}$$

with three dimensionless governing parameters

$$\lambda = \frac{k_2}{k_1}, \qquad \kappa = \frac{k_1}{k_{-1}}, \qquad \rho = \frac{k_3 C_{\mathrm{P}}^0}{k_1}. \tag{S57}$$

The semi-infinite boundary conditions in dimensionless form become

$$\lim_{y\to\infty} p = 1, \quad \lim_{y\to\infty} q = \lim_{y\to\infty} b = \lim_{y\to\infty} c = 0. \tag{S58}$$

At the electrode surface, the dimensionless boundary conditions are

$$p(0) + q(0) + b(0) + c(0) = 1, \tag{S59}$$

$$\frac{dp}{dy}(0) + \frac{dq}{dy}(0) + \frac{db}{dy}(0) + \frac{dc}{dy}(0) = 0. \tag{S60}$$

Additionally, the dimensionless plateau current is given by

$$\psi = -\frac{dq}{dy}(0). \tag{S61}$$

The final homogeneous electron transfer step ($\mathbf{E}'$) is typically rapid, such that we set the parameter ρ to a large constant value $\rho = 10^9$ and obtain a two-dimensional system in terms of λ and κ:

$$X = \log\kappa, \qquad Y = \log\lambda. \tag{S62}$$

Finally, the current function for this system is defined as

$$\Psi = \log\left(\psi\left(\kappa, \lambda\right)\right). \tag{S63}$$

5 Curvature analysis of nonlinear behavior

Transitioning across certain zone boundaries induces abrupt changes in the observed kinetic behavior. As described in the main text, this is due to the effect of nonlinear terms in the governing equations. We find that such transitions can be identified by narrow regions in the parameter space displaying high curvature. Here we demonstrate how these observed high-curvature transitions correspond to nonlinear kinetics.

For the case of mediate enzymatic catalysis (Section 4.2), one such transition occurs as $X = \log\gamma$ is increased in the upper left corner of the zone diagram, where $Y = \log\kappa$ is large (Figure S5a). The curvature in the transition zone traversed in this example is relatively large (signaled by the bright yellow region). We start by examining the dimensionless governing equation

$$\frac{d^2q}{dy^2} = \frac{\kappa^2 qs}{\gamma q\left(1 + \mu s\right) + s} = 0. \tag{S64}$$

Since κ is large, we can resale the spatial variable according to $\tilde{y} = y\kappa$, leaving

$$\frac{d^2q}{d\tilde{y}^2} - \frac{qs}{\gamma q\left(1 + \mu s\right) + s} = 0. \tag{S65}$$

Starting with the case where $0 < \gamma \ll 1$, we examine the denominator

$$\gamma q + \gamma\mu qs + s. \tag{S66}$$

Considering $\mu = \mathcal{O}(1)$, the dominate balance is

$$\gamma q \sim \gamma\mu qs \ll s, \tag{S67}$$

and the terms containing γ are negligible in front of s. This reduces the governing equation to

$$\frac{d^2q}{d\tilde{y}^2} - q = 0. \tag{S68}$$

This is a linear equation in q, and corresponds to the behavior on the far left of the zone diagram (red dot, Figure S5a). Now by increasing γ, all three terms in the denominator balance, such that

$$\gamma q \sim \gamma\mu q s \sim s. \tag{S69}$$

As a result, the governing differential equation will contain quadratically nonlinear terms qs, and the translation on the zone diagram between $0 < \gamma \ll 1$ and $\gamma = \mathcal{O}(1)$ (represented by the arrow in Figure S5a) exhibits an abrupt crossing from linear to nonlinear behavior. This transition can be identified by the large increase in curvature.

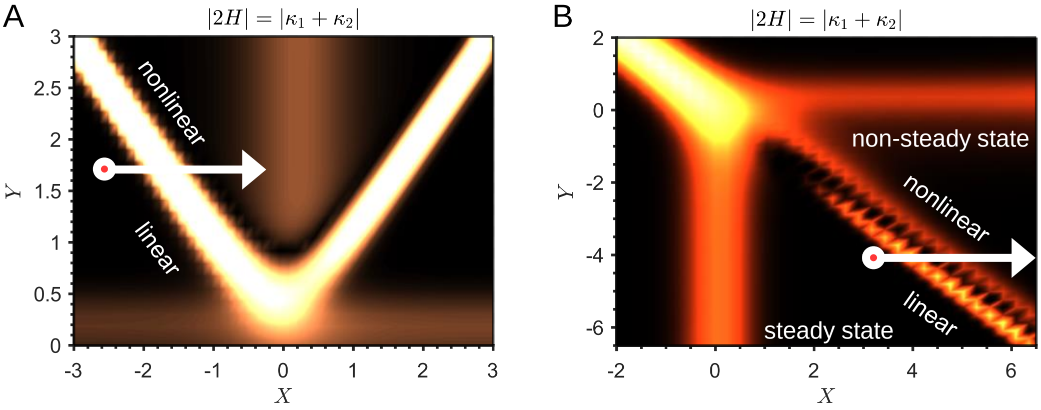

Figure S5. Analyzing the transition from linear to nonlinear kinetics with examples from (a) mediated enzymatic catalysis in a film and (b) homogeneous CO_2 reduction (**ECCE′**). The zone diagrams as displayed as heat maps of the absolute value of the mean curvature $|2H|$. The example translations in the zone diagrams, indicative of triggering the onset of nonlinear behavior, are indicated by the bold arrow.

A nonlinear transition was also observed in the **ECCE′** mechanism for CO_2 reduction (Section 4.3) [9]. Beginning in the bottom right of the zone diagram (where the parameter $Y = \log\lambda$ is small), increasing the parameter $X = \log\kappa$ led to a transition where the intermediate C is first under a steady state and then rapidly progresses to a situation where C accumulates in the diffusion layer (non-steady state). This sharp transition can be identified by the high curvature (see the arrow in Figure S5b) and by the extremely narrow transition region. Examining the system of equations reveals this behavior corresponds to activating nonlinear reaction terms, triggered by translation along the X-axis. Consider the governing equations

$$\frac{d^2q}{dy^2} - q + \kappa^{-1}b - \rho qc = 0, \tag{S70}$$

$$\frac{d^2b}{dy^2} + q - (\kappa^{-1} + \lambda)b = 0, \tag{S71}$$

$$\frac{d^2c}{dy^2} + \lambda b - \rho qc = 0. \tag{S72}$$

Savéant and Costentin have previously shown that when $0 < \lambda \ll 1$ and $\kappa = \mathcal{O}(1)$, the intermediate C is behaves at steady state (its concentration is very small and constant over the diffusion

layer) [9]. If C is at steady state, its total concentration will scale as ρ^{-1} where $0 < \rho^{-1} \ll 1$. Let $\tilde{c} = c\rho$, yielding

$$\frac{d^2 q}{dy^2} - q + \kappa^{-1} b - q\tilde{c} = 0, \tag{S73}$$

$$\rho^{-1} \frac{d^2 \tilde{c}}{dy^2} + \lambda b - q\tilde{c} = 0. \tag{S74}$$

Leveraging now the small parameter ρ^{-1}, we introduce asymptotic expansions [1, 2] in powers of $\epsilon := \rho^{-1}$:

$$q \sim q_0 + \epsilon q_1 + \epsilon^2 q_2 + \dots, \tag{S75}$$

$$\tilde{c} \sim \tilde{c}_0 + \epsilon \tilde{c}_1 + \epsilon^2 \tilde{c}_2 + \dots, \tag{S76}$$

$$b \sim b_0 + \epsilon b_1 + \epsilon^2 b_2 + \dots. \tag{S77}$$

Substituting these expansions into the differential equations and retaining only the leading order $\mathcal{O}(1)$ terms, leaves $\lambda b_0 = q_0 \tilde{c}_0$. A final substitution results in a linear system of two coupled equations as reported previously [9]:

$$\frac{d^2 q_0}{dy^2} - q_0 + (\kappa^{-1} - \lambda) b_0 = 0, \tag{S78}$$

$$\frac{d^2 b_0}{dy^2} + q_0 - (\kappa^{-1} + \lambda) b_0 = 0. \tag{S79}$$

It was shown that as κ increases, C is no longer at steady state [9]. In this situation, we must rescale the spatial variable. Let $\tilde{y} = y\sqrt{\rho}$, giving

$$\frac{d^2 q}{d\tilde{y}^2} - \epsilon q + \epsilon \kappa^{-1} b - qc = 0, \tag{S80}$$

$$\frac{d^2 c}{d\tilde{y}^2} + \epsilon \lambda b - qc = 0. \tag{S81}$$

Substituting the same asymptotic expansions and retaining only the lowest order terms results in

$$\frac{d^2 q_0}{d\tilde{y}^2} - q_0 c_0 = 0, \tag{S82}$$

$$\frac{d^2 c_0}{d\tilde{y}^2} - q_0 c_0 = 0. \tag{S83}$$

These two differential equations both contain the quadratically nonlinear term $q_0 c_0$, giving rise to the abrupt transition and sharp increase in the curvature as C transitions from a steady to non-steady state.

6 Methods

6.1 Numerical methods

The dimensionless systems described above were discretized and solved using finite difference method [10, 11]. These were first implemented as time-dependent systems of partial differential

equations, and solutions obtained this way were then time marched to yield the steady-state plateau current. In all cases, the time variable was non-dimensionalized with a general experimental timescale θ, where we define dimensionless time as $\tau = t/\theta$. This was varied in each case to ensure a pseudo-steady solution was reached.

The models including a heterogeneous film as the spatial domain (electrocatalytic films, Section 4.1; and mediated enzymatic films, Section 4.2) were implemented numerically using MATLAB's `pdepe` solver. Fluxes were calculated using the inbuilt `pdeval` function. A double exponential grid was used to discretize the spatial domain on the interval $y \in [0, 1]$ (Figure S6, left column).

In the case of the homogeneous mechanism (Section 4.3), a backwards implicit finite difference scheme was used on an unconstrained exponential grid (Figure S6, right column). Due to the presence of nonlinear reaction terms a Newton-Raphson root-finding method was employed [11]. At each time step, the system of discritized equations, represented by a vector $\mathbf{F}$, were zeroed. The known set of concentration values from the previous time step $\mathbf{x}$ were used as an initial guess, and a new set of concentration values $\mathbf{x} + \delta\mathbf{x}$ were calculated according to

$$\mathbf{F}(\mathbf{x} + \delta\mathbf{x}) = \mathbf{F}(\mathbf{x}) + \mathbf{J} \cdot \delta\mathbf{x}, \tag{S84}$$

where $\mathbf{J}$ is the Jacobian matrix, and $\delta\mathbf{x}$ is a vector containing correction terms to be determined. With $\mathbf{F}(\mathbf{x} + \delta\mathbf{x}) = 0$, the matrix equation to be solved becomes

$$\mathbf{J} \cdot \delta\mathbf{x} = -\mathbf{F}(\mathbf{x}). \tag{S85}$$

This process was iterated until the solution converged to a tolerance of 10^{-6}. The current was calculated using two-point approximation to the first spatial derivative with a step size $\Delta y = 10^{-5}$.

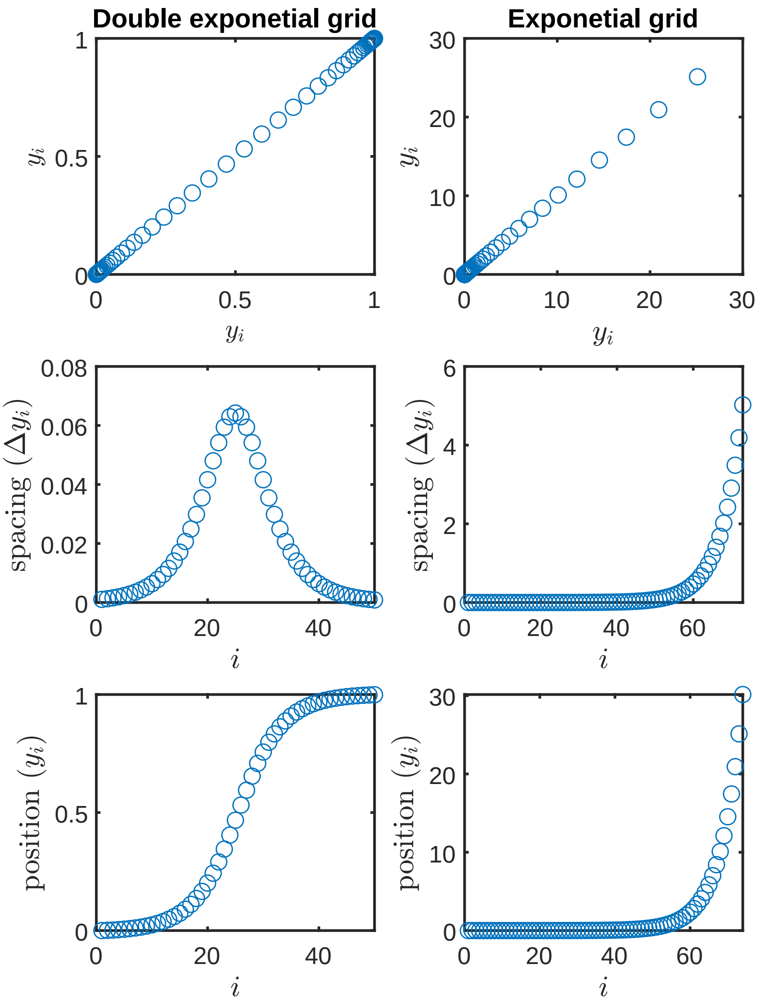

Figure S6. Spatial grids employed in the numerical solutions [10]. The grid spacing Δy_i and the position y_i are plotted as function of grid index i. For the models including a film domain, a double exponential grid (left column) was used to provide a high density of grid points at each interface. For the homogeneous model on a semi-infinite domain a simple exponentially expanding grid was used (right column).

Author Contributions

B.A.J. conceived the research, led the project, developed the theory, designed and conducted the case studies (including the analytical modeling and numerical simulations), wrote the original draft, and reviewed and edited the manuscript. N.P. provided feedback on the review and editing of the manuscript.

References

(1) Bender, C. M.; Orszag, S. A., *Advanced Mathematical Methods for Scientists and Engineers I: Asymptotic Methods and Perturbation Theory*, 1st ed.; Springer New York, NY: 1999.

(2) Holmes, M. H., *Introduction to Perturbation Methods*, 2nd ed.; Springer New York, NY.

(3) Kristopher, T., *Differential Geometry of Curves and Surfaces*; Springer Cham: 2016.

(4) Costentin, C.; Savéant, J.-M. *ChemElectroChem* **2015**, *2*, 1774–1784.

(5) Andrieux, C. P.; Dumas-Bouchiat, J. M.; Savéant, J.-M. *J. Electroanal. Chem.* **1984**, *169*, 9–21.

(6) Thiele, E. W. *Ind. Eng. Chem.* **1939**, *31*, 916–920.

(7) Andrieux, C.; Savéant, J. *J. Electroanal. Chem. Interf. Electrochem.* **1982**, *134*, 163–166.

(8) Bartlett, P.; Pratt, K. *J. Electroanal. Chem.* **1995**, *397*, 61–78.

(9) Costentin, C.; Savéant, J.-M. *ACS Catal.* **2018**, *8*, 5286–5297.

(10) Britz, D.; Strutwolf, J., *Digital Simulation in Electrochemistry*, 4th ed.; Springer International Publishing: 2016.

(11) Compton, R. G.; Laborda, E.; Ward, K. R., *Understanding Voltammetry: Simulation of Electrode Processes*; Imperial College Press: 2013.
